# Race and other sociodemographic categories are differentially linked to multiple dimensions of interpersonal-level discrimination: Implications for intersectional, health research

**DOI:** 10.1371/journal.pone.0251174

**Published:** 2021-05-19

**Authors:** Danielle L. Beatty Moody, Shari R. Waldstein, Daniel K. Leibel, Lori S. Hoggard, Gilbert C. Gee, Jason J. Ashe, Elizabeth Brondolo, Elias Al-Najjar, Michele K. Evans, Alan B. Zonderman

**Affiliations:** 1 Department of Human Services Psychology, University of Maryland, Baltimore, Maryland, United States of America; 2 Division of Gerontology and Geriatric Medicine, Department of Medicine, University of Maryland School of Medicine, Baltimore, Maryland, United States of America; 3 Geriatric Research Education and Clinical Center, Baltimore VA Medical Center, Baltimore, Maryland, United States of America; 4 Department of Psychology, Rutgers University, New Brunswick, New Jersey, United States of America; 5 Department of Community Health Sciences, University of California, Los Angeles, Los Angeles, California, United States of America; 6 Department of Psychology, St. John’s University, Queens, New York, New York, United States of America; 7 Department of Mathematics and Statistics, University of Maryland, Baltimore County, Baltimore, Maryland, United States of America; 8 Laboratory of Epidemiology and Population Sciences, National Institute on Aging, National Institutes of Health, Baltimore, Maryland, United States of America; Montclair State University, UNITED STATES

## Abstract

**Objectives:**

To examine whether intersections of race with other key sociodemographic categories contribute to variations in multiple dimensions of race- and non-race-related, interpersonal-level discrimination and burden in urban-dwelling African Americans and Whites.

**Methods:**

Data from 2,958 participants aged 30–64 in the population-based Healthy Aging in Neighborhoods of Diversity across the Life Span (HANDLS) study were used to estimate up to four-way interactions of race, age, gender, and poverty status with reports of racial and everyday discrimination, discrimination across multiple social statuses, and related lifetime discrimination burden in multiple regression models.

**Results:**

We observed that: 1) African Americans experienced all forms of discrimination more frequently than Whites, but this finding was qualified by interactions of race with age, gender, and/or poverty status; 2) older African Americans, particularly African American men, and African American men living in poverty reported the greatest lifetime discrimination burden; 3) older African Americans reported greater racial discrimination and greater frequency of multiple social status-based discrimination than younger African Americans; 4) African American men reported greater racial and everyday discrimination and a greater frequency of social status discrimination than African American women; and, 5) White women reported greater frequency of discrimination than White men. All *p*’s < .05.

**Conclusions:**

Within African Americans, older, male individuals with lower SES experienced greater racial, lifetime, and multiple social status-based discrimination, but this pattern was not observed in Whites. Among Whites, women reported greater frequency of discrimination across multiple social statuses and other factors (i.e., gender, income, appearance, and health status) than men. Efforts to reduce discrimination-related health disparities should concurrently assess dimensions of interpersonal-level discrimination across multiple sociodemographic categories, while simultaneously considering the broader socioecological context shaping these factors.

## Introduction

In the United States (U.S.), discrimination continues to function chiefly as a consequence of dominant versus nondominant group membership, with minority categorizations delineated in accordance with sociohistorically established classifications [1, 2]. Arguably, race is the most impactful minority sociodemographic category in the U.S., driving striking and protracted economic, social, and health disparities among most racial minorities compared with Whites [3–5]. Most research on discrimination has examined the correlates of these experiences in one-on-one or interpersonal interactions and has focused on African Americans [e.g., 6–10]. The emphasis on African Americans is likely because of their distinct experiences—their unique passage to the U.S. and subsequent institutionalized enslavement and oppression; de jure and modern de facto discrimination; and their disproportionate burden of poor health [1, 3, 4, 10, 11]. However, Whites—the dominant racial group in the U.S.—are increasingly reporting interpersonal-level discrimination, which has been linked to poor health (e.g., [12]). While it is unsurprising that discrimination has adverse health consequences [13], we lack a rudimentary understanding of how the interactive association between race and other key sociodemographic categories is related to reports of interpersonal-level discrimination for African Americans and Whites.

### Conceptualizing racism and racial discrimination

Racism is defined as “a system of oppression based on racial/ethnic group designations in which a pervasive ideology of racial superiority and inferiority provides the foundation for structural inequalities, intergroup conflict, discrimination, and prejudice” [4 p396]. Accordingly, racial discrimination—defined as actions by members of dominant racial groups that have negative or differential effects on members of nondominant racial groups [14]—is the behavioral manifestation of racism. Racial discrimination emanates from a system of oppression, based on power asymmetries that allow the dominant group to maintain access to unearned privileges, opportunities, and resources [1]. As we discuss the racism and discrimination—we use the sets of terms “African American” and “Black” and “White American” and “White,” respectively, to reflect these racialized groupings as used in the related literature.

### Intersecting sociodemographic categories and dimensions of discrimination

When considering race, other sociodemographic categories for which marginalized identities are established may also affect experiences with discrimination. For many, race is experienced in tandem with other personal characteristics, including age, gender, and socioeconomic status (SES). For instance, the experiences of an older African American male with a lower SES may markedly differ from those of a younger White male with a higher SES. Thus, it may be insufficient to examine the “raced-experience” of African Americans without also considering age, gender, and SES—as each of these characteristics encompasses socially constructed meanings that may influence an individual’s perceived social standing and interpersonal-level interactions. As such, simultaneous consideration of an individual’s different minority or non-minority statuses may offer greater insight than simply studying these statuses in isolation. Indeed, it is plausible that each respective sociodemographic membership uniquely contributes to their experiences of discrimination, as well as towards the overall cumulative experience and impact of these events.

A current gap regarding interpersonal-level discrimination research is that this multidimensional construct is traditionally assessed through emphasis on one aspect or domain of discriminatory experiences [15]. Specifically, while some research does assess several dimensions concurrently [e.g., 16–21], most research characterizing discrimination has not considered multiple facets of interpersonal discriminatory acts in a comprehensive manner, including their types (e.g., generic versus status specific [race- or gender-related]), context (e.g., workplace, court setting), frequency (e.g., daily, lifetime), form of threat (e.g., socio-emotional, physical), explicitness (e.g., covert versus overt), and consequences (e.g., posed obstacles, blocked opportunities). This is a particular limitation in descriptive discrimination research.

Understanding the differential patterning of these dimensions of discrimination as a function of an individual’s concurrent sociodemographic categories may enable greater precision in elucidating linkages to mental and physical health endpoints. This approach may also allow health disparities researchers to uncover previously obscured subgroups with a high risk of experiencing discrimination and who, in turn, face greater health vulnerabilities.

### Sociodemographic variations in exposure to dimensions of discrimination

…Racism will always be a part of the world, a part of America…Hate in America, especially for African-Americans, is living every day…No matter how much money you have, no matter how famous you are…being black in America is tough…And we got a long way to go, for us as a society and for us as African-Americans, until we feel equal in America. [22]

–LeBron James

LeBron James made this statement in 2017, hours after his home was vandalized with racist graffiti. His response echoes the experience of many African Americans in the U.S.: racism is unyielding and pervasive, regardless of how much they accomplish. Indeed, over the last several years, a constant and growing barrage of national news reports have illuminated the myriad occurrences of racial discrimination experienced by African American women, men, and children across the country. White bystanders have requested unnecessary police intervention in situations where African Americans are engaging in routine, everyday activities such as bird watching, jogging, swimming in community pools, operating lemonade stands, entering their apartments, moving into new single-family homes, hosting neighborhood barbeques, or transacting business with their bank teller at a local financial institution [12, 23–36]. Paralleling these news reports is an uptick in anti-Black hate crimes documented by the Federal Bureau of Investigation across 2017 and 2018 [24, 25]. Several empirical studies support these reports of racial discrimination against African Americans and also explore how other sociodemographic categories (e.g., age, gender, and SES) intersect with race and contribute to discriminatory experiences [37–39].

### Characterizing discrimination in blacks

Across decades of social science research, African Americans consistently self-report the highest levels of race-related and general interpersonal discrimination across major life domains. Indeed, 80 to 100% of African Americans report lifetime exposure to racial discrimination in daily life [26–32]. In one longitudinal study, 90% of African Americans reported persistent racial discrimination at work; in the marketplace; when seeking employment, housing, or medical care; and when engaging with law enforcement and the judicial system across a seven-year span [40]. African Americans also report the highest levels of *everyday discrimination* [12], which includes day-to-day minor insults without attribution of cause (e.g., race, age, gender, SES). While racial differences in the overall lifetime burden of discrimination remain unclear, reports overwhelmingly demonstrate that African Americans experience discrimination more consistently throughout their lives as compared to other racial/ethnic groups [e.g., 33–35].

Reports of interpersonal-level discrimination vary by age, gender, and SES, and these variations may also be modified by race. Regarding age, Gee et al., [36] posit that experiences with discrimination may be shaped by historical events across one’s life. For example, racial minorities living during particularly polarizing racialized periods, such as the Civil Rights era (i.e., 1954 to 1968), might be expected to report more accumulative discriminatory experiences than their younger peers or Whites [41]. However, findings are mixed with some studies showing that older African Americans report more overall and racial discrimination [42–45] and some reporting no differences in reported discrimination according to age (e.g., [27, 46–48]). Yet, there are also studies which demonstrate that younger African Americans report more overall and racial discrimination [12, 49, 50]. Notably, Gee and colleagues [36] emphasize the role of historical, racialized periods in shaping exposure to discrimination; a perspective which may shed light on why younger generations of African American adults would also report more of these experiences similar to older African Americans. Indeed, the accumulation of contemporary sociopolitical and structural discrimination, growing awareness of police-involved killings of unarmed African Americans, and the related wave of protests against social injustice over the last decade, likely serve as an unwelcome backdrop for the interpersonally discriminatory experiences that the younger generation faces on a daily basis. With regard to differences in exposure by age between African Americans and Whites, there is some evidence that older African Americans may report greater everyday discrimination than older Whites (e.g., [51]). Overall, the interplay between age and various types of discrimination experienced by African Americans remains poorly understood and has not been explored in comparison to White experiences.

Data regarding gender differences in overall and racial discrimination are also mixed [5–8]. Most studies have focused on African Americans and have examined gender differences within, rather than between, racial groups. Most, but not all, of these studies show that African American men report higher levels of overall and racial discrimination compared with African American women [12, 52–54]. However, existing discrimination measures may not fully capture gender differences [40, 55] or encompass the intersectional nature of race and gender reflecting the experiences of African Americans [39, 56, 57]. African American men face pervasive, widely publicized, discriminatory events and are subject to hostile encounters with law enforcement and the judicial system [58–60]. However, African American women also face chronic, unfair, hostile, and even deadly encounters across their lifetimes, which are lesser known, but are beginning to draw attention across national media platforms [e.g., 61, 62] and in empirical studies [e.g., 55]. Because their doubly marginalized status is often unaccounted for, empirical studies may not fully capture the discrimination that African American women face; therefore, examining how race interacts with gender and other key sociodemographic categories (e.g., SES) in relation to discrimination may be a step toward providing greater clarity on their experiences. African American men and women occupy marginalized statuses—both are from a minority race (African American) and African American women are also from a devalued gender grouping [63–67]. These sociodemographic categories largely shape their ascribed level of threat and access to opportunities and benefits, and consequently, the types of discriminatory treatment they receive.

Many studies report that for African Americans, higher SES—whether measured by poverty level, income, or education—is associated with increased reports of various forms of self-reported discrimination [5, 33, 55, 68], especially for men [69, 70]. While some studies have reported no variation by SES in discriminatory experiences (e.g., [27]), others have shown that lower SES is associated with more frequent experiences of everyday or racial discrimination (e.g., [26, 41]) by African Americans and Whites. Few studies have explored whether African Americans face different types of discrimination as a function of their SES [47, 71, 72]. According to one study, African Americans with lower incomes and less education may face race-related stigma and physical threat or aggression, whereas those with higher incomes may face more racial discrimination in the workplace, indicating that SES variations may confer unique discriminatory experiences for African Americans [73]. Although studies of the interplay of SES and discrimination for Whites are limited, evidence suggests that those with greater poverty exposure and less education are more likely to report racial discrimination than their peers with a higher SES based on these indicators [33]. Therefore, SES differences in discrimination may vary by the metric used to measure SES and may also depend on the dimension of discrimination considered [47]. Notably, scholars have demonstrated that indicators of SES, including education and poverty, are not proxies for each another, nor are they interchangeable, but capture distinct information on SES [74]. Accordingly, we analyze the relationship of both poverty and education independently in relation to our outcomes of interest in this study.

### Characterizing discrimination in whites

Embedded within the conceptualization of racism in the U.S. is the fundamental understanding that members of the dominant racial group, White Americans, are not, nor can they be, subject to systematic racism [1, 75]. Still, consideration must be given to the complex, intersecting histories of race, ethnicity, and Whiteness as related to discrimination in this country. For instance, prior to their acceptance into the broader racial grouping of *White*, some Euro-ethnic groups, such as Jewish and Greek people, experienced pervasive structural-level discrimination, from the “establishment” group, White Anglo-Saxon Protestants [76]. Against this backdrop, in contemporary reports, Whites are increasingly identifying their group as targets of racial discrimination. For instance, a 2017 nationally representative survey of Whites indicated that 55% believe anti-White discrimination exists, and 43% of non-Hispanic Whites from another survey believed racism against their group is widespread [26, 77]. However, in contrast to perceiving discrimination against their group [78], few Whites report personally experiencing racial discrimination. In a 2016 Pew poll, 30% of White respondents reported encountering racial discrimination, with only 2% identifying these as regularly occurring experiences [79]. These patterns of self-reported discrimination demonstrate that all groups might experience out-group threat, yet only some encounter discrimination on a daily basis. Notably, ethnic membership among Whites may contribute to variations in their experiences with discrimination. At the same time, these experiences in Whites are not fundamentally tied to an underlying system of structural racism. This foundational aspect of how race functions in the U.S. for Whites as compared to Blacks, has wholly shaped their differing experiences of racism and discrimination, including interpersonal-level racial/ethnic events.

While interpersonal discrimination related to race or ethnicity can happen to anyone, some researchers suggest that these reports in Whites may reflect other underlying factors central to the American cultural milieu. In this regard, several reports demonstrate growing concern and discomfort among Whites related to the rising themes of inclusivity and equity [80–85] and shifting demographics in the U.S., as the U.S. Census Bureau predicts racial/ethnic minorities will comprise the majority of the nation’s population by 2044 [86]. These concerns may emanate from a zero-sum perspective—where members of a dominant group feel they “lose” if a nondominant group “wins” (e.g., by achieving equity and/or comprising the majority of the nation’s population), thereby producing negative racial sentiment in the dominant group [78, 80]. Indeed, per traditional social psychological theory, perceived alterations to the traditional social hierarchy and related privileges would be deemed threatening for any majority group [87]. Taken together, these factors may inspire negative sentiment and contribute to increased perceptions of race-based discrimination among Whites.

These factors (concerning the possible underlying reasons for these reports) notwithstanding, clearly, *perception matters*. Many studies show that the interpersonal experience of discrimination is real and valid for the target, regardless of the underlying cause, and that these experiences can impact biological processes that contribute to poor health outcomes [5, 8, 9]. Indeed, preliminary but growing evidence shows that discrimination is associated with negative health effects among Whites who affirm having these experiences [17, 20, 21, 50, 88].

Within Whites, intersecting gender and SES social identities may uniquely shape their reports of discrimination. For example, White women, given their juxtaposition of White racial privilege and devalued gender status, may experience particular forms of discrimination [e.g., see 89]. For instance, in a past national survey, White respondents (39.2%) were most likely to identify gender as the primary cause of discrimination they experienced [12]. Whites with a lower SES may also have unique discriminatory experiences or perceptions about discrimination [e.g., 90, 91]. Whether and how age, gender, and SES may shape Whites’ experiences with discrimination remains unknown. Characterizing reports of discrimination by Whites, particularly in the context of other key sociodemographic factors, can help clarify how these experiences negatively impact their health. In sum, comprehensive examination of these linkages in Whites—where they have been understudied—and African Americans, may shed much needed light on how age, gender, and SES uniquely or similarly shape their experiences of discrimination.

### Guiding integrative theoretical framework

Our study draws on racial stratification theory [92, 93] and the racism-related stress [1], intersectionality [94, 95], and health disparities frameworks through considering four key tenets. First, macro-level U.S. sociohistorical dynamics have created a bifurcated grouping that deems Blacks as lowly valued and less desirable than Whites and other racial/ethnic groups [3, 96]. This culturally held understanding of the racial hierarchy consciously and unconsciously influences interpersonal interactions for African Americans and Whites [97, 98]. Although every discriminatory event that African Americans experience is not explicitly attributed to their race, their highly marginalized minority group membership still produces greater discriminatory exposure which may be characterized as race- or non-race-related [1]. Second, an individual likely experiences their social statuses as intersecting identities [95]. In this regard, intersectionality is a theoretical framework forwarded by Kimberly Crenshaw [95], a Black feminist scholar that posits that multiple social categories—such as race, age, gender, and SES—intersect at the individual-level to reflect established structural-level systems of privilege and oppression. Thus, considering race alongside other sociodemographic categories may allow greater understanding of their contributions to the experience of discrimination [7, 56, 94, 95, 99–101]. Notably, this approach may shed light on the *intersectional paradox* [56], wherein an individual simultaneously occupies low and high social statuses (e.g., African American men or White women). Third, occupying a lower social status or multiple marginalized statuses may uniquely shape the types, contexts, frequency, and severity of discriminatory experiences. Finally, applying an integrative framework allows us to parse out the complex linkages between social statuses and interpersonal-level discrimination. This may be an important but overlooked step that may aid the resolution of equivocal findings in health disparities research related to racially disproportionate health outcomes and the contribution of social and psychological determinants of health. Indeed, disproportionate, chronic experiences with discrimination based on multiple aspects of one’s social identity may contribute to negative biopsychosocial sequelae via sustained psychological and physiological wear and tear (e.g., allostatic load).

### Goals of current study

This study examines the complex linkages among key intersecting sociodemographic categories, specifically, race, age, gender, and SES, in relation to various dimensions of interpersonal-level discrimination in a cohort of socioeconomically diverse, urban-dwelling, middle-aged to older African American and White adults. Specifically, we propose that while both racial groups may experience discrimination, African Americans will report experiencing more interpersonal-level discrimination than Whites. Further, we expect different dimensions of discrimination, particularly, social-status-specific discrimination (e.g., racial) and non-social status specific discrimination (e.g., everyday, chronic experiences), as well as the overall burden of discrimination to vary across distinct, intersecting sociodemographic categories. While complex variations in the frequency, severity, and sources of discrimination likely occur along the lines of race, age, gender, and SES, most prior studies that examine discrimination as an endpoint adjust for one or more of these sociodemographic factors (e.g., [47, 49, 102]) without examining their potential interactions (for exceptions, see [53, 55]). Therefore, our exploratory investigation seeks to uncover how different combinations of these key sociodemographic categories may contribute to an individual’s experience of multidimensional discrimination. In turn, as discriminatory experiences are linked to a range of negative mental and physical health outcomes [9], our study will explore how exposure to discrimination is characterized by sociodemographic characteristics, which may also inform our understanding of associations between multidimensional discrimination and health endpoints. Thusly, the primary aim of the present study is to explicitly test the hypothesis that African American race—a key sociodemographic characteristic—will be associated with greater self-reported discrimination. Further, to address inconsistencies in the literature, we test up to 3-way interactions between race and age, gender, and SES in relation to the four concurrently-assessed measures of interpersonal-level discrimination (racial discrimination, discrimination across multiple social statuses, everyday discrimination, and lifetime discrimination burden).

## Methods

### Participants and procedure

Healthy Aging in Neighborhoods of Diversity across the Life Span (HANDLS) is a planned 20-year, prospective, population-based, longitudinal study, designed to investigate the associations among race, age, gender, and SES, and risk factors for health disparities [103]. An area probability sampling strategy stratified by 5-year age bands (30–64), sex, self-identified race (White or African American), and household income below or above the Federal household-size poverty level was utilized to determine the sample. HANDLS participants are a fixed cohort of 3,720 urban-dwelling, African American and White participants who were between 30 and 64 years old at baseline (2004–2009). Participants were drawn from an area probability sample of thirteen neighborhoods (contiguous census tracts) in the city of Baltimore, Maryland. In-home visits and mobile research vehicles were utilized to collect data. All participants provided written informed consent. The study protocol was approved by the Institutional Review Board at the National Institute of Environmental Health Sciences.

At initial selection for participation in HANDLS, participants were excluded if pregnant, within 6 months of cancer treatment, diagnosed with AIDS, limited in ability to provide written informed consent, unable to provide valid government-issued identification, or currently without a verifiable address. The current sample included 2,958 adults, of whom 1,924 were African American and 1,034 were White. However, the number of participants included in each analysis varied slightly because of missing data points on different discrimination measures.

### Measures

#### Demographic characteristics

Participants’ age, race (0 = White, 1 = African American), sex (0 = woman, 1 = man), gender (0 = female, 1 = male), annual household income, and educational attainment were assessed. Nine participants self-identified their ethnicity as Hispanic, all of whom also self-identified their race as White. Self-identified gender was determined based upon participant reports that their gender differed from their biological sex. Three participants identified as women whose biological sex assigned at birth was male. Poverty status was defined as annual household income (adjusted for household size) above (0) or below (1) 125% of the 2004 Health and Human Services poverty guidelines. Educational attainment was categorized into three levels (0 = less than high school, 1 = high school diploma or GED, or 2 = more than high school).

#### Discrimination measures

***Racial discrimination*** was assessed with a summed, six-item measure initially used in a large prospective, epidemiological cohort study [104] and subsequently included in the Experiences of Discrimination scale, which has adequate internal consistency (α = 0.74) and test-retest reliability (0.70; [105]). The measure included questions about whether individuals experienced racial discrimination at school, when seeking employment, at work, while getting housing, when getting medical care, or from police or in courts [106]. Respondents replied *Yes* (1) or *No* (0) to each item. Possible scores ranged from 0–6, with higher values indicating more racial discrimination. In our study population, this scale had strong internal consistency among African Americans (α = 0.82) and Whites (α = 0.77).

***Discrimination across multiple social statuses*** was assessed using a summed, ten-item measure adapted from a previous measure of discrimination in healthcare settings [104, 107]. The measure included, “Overall, how much have you experienced prejudice or discrimination because of…” gender, race, ethnicity, income, age, religion, physical appearance, sexual orientation, health status, and disability. Participants responded on a 4-point scale ranging from 1 *(not all all*) to 4 (*a lot*). Possible scores can range from 10 to 40, with higher scores indicating more forms of discrimination were experienced more frequently. In our study population, this scale had strong internal consistency among African Americans (α = 0.84) and Whites (α = 0.74).

The ***Everyday Discrimination*** scale [50, 105] is a summed, nine-item measure assessing the frequency of day-to-day experiences of bias without attribution to race or ethnicity (e.g., “being treated with less courtesy”). Participants responded on a 6-point scale ranging from 1 (*almost every day*) to 6 (*never*). Responses were reverse scored and summed. Possible scores can range from 9–54, with higher scores indicating greater everyday discrimination. This scale has been shown to have strong internal consistency among African Americans and Whites (α = 0.88; [50]) and was similarly strong in our sample (α = 0.83 for African Americans and Whites when examined separately).

***Lifetime discrimination burden*** was assessed with a summed, two-item measure. These items have been used in large-scale epidemiologic studies [104, 108, 109]: (1) “Overall, how much has discrimination interfered with you having a full and productive life?” and “Overall, how much harder has your life been because of discrimination?” Participants responded on a 4-point scale ranging from 1 (*not at all*) to 4 (*a lot*) for each item. Possible scores can range from 2–8, with higher scores indicating greater lifetime discrimination burden. In the present sample, these two items were strongly correlated among African Americans, *r* = 0.74, *p* < .001, and Whites, *r* = 0.79, *p* < .001.

### Statistical plan

R version 1.2.1335 [110] was used for statistical analyses. Preliminary data analysis revealed positively skewed distributions and residual plots for all discrimination measures except everyday discrimination. Logarithmic- and square-root-transformations failed to correct the skewness of the distributions or residuals. Therefore, generalized linear models using an inverse Gaussian link function were run to examine moderating effects of age, gender, and poverty status on associations between race and the four discrimination outcomes. Models were run separately for each of the four discrimination measures. Analyses began with fully adjusted models, which contained all two-, three-, and four-way interactions and main effects of race, age, gender, and poverty status. If the four-way interaction effect was statistically significant (*p*-value < 0.05), the full model was retained. Conversely, if the four-way interaction effect was not significant, that interaction was removed, and the analysis proceeded through a backward elimination procedure [111]. This was repeated with higher order interactions that did not show statistically significant relationships in a stepwise fashion, beginning with the four-way interaction and followed by all three-way and two-way interactions in subsequent steps. This procedure continued until the highest-order interaction effect(s) that included race was identified, at which point the model was retained. All significant interactions were probed for simple effects and plotted to assist with interpretation. Models were also tested using the three-level education measure in lieu of poverty status to determine whether and how this SES indicator uniquely moderated the association of race with age and gender in relation to the discrimination outcomes. Finally, to determine whether Hispanic ethnicity influenced the results, analyses were rerun after excluding the nine participants who identified as Hispanic (see S2 File for detailed descriptions of these analyses and findings).

Adjustment for multiple comparisons were not made for several reasons, as outlined by other epidemiological researchers [112, 113]. Some of the primary reasons were because while these adjustments may reduce Type I errors, the likelihood of Type I errors cannot decrease without increasing the likelihood of Type II errors [114, 115], and also because adjusting for multiple comparisons may deem truly important group differences as nonsignificant. In addition, multiple comparison adjustments may also be problematic because their use implies that interpretation of a given effect is conditional on the number of tests performed, rather than on what the data demonstrate [112, 113]. Thusly, 95% confidence intervals were included for all effects in our regression model tables (see Tables 2 and 3) to provide information about the practical significance of our results.

## Results

African Americans were significantly more likely to have household incomes below 125% of the poverty line and had higher scores on all discrimination measures than Whites (*p*’s < .001; Table 1). In addition, a significantly greater proportion of Whites identified as women (56.6%) compared to the proportion of African Americans who identified as women (52.6%; χ^2^(1) = 4.28, *p* = .038). All discrimination measures were positively correlated, demonstrating moderate (*r* = 0.35) to high (*r* = 0.62) correlations with one another (S1 Table).

**Table 1:** Participant characteristics in the overall sample and stratified by race.

Variable	African American (*n* = 1,924)	White (*n* = 1,034)	sig.	All (*N* = 2,958)
Age	48.25 (±9.26)	47.92 (±9.50)		48.13 (±9.34)
Women, %	52.6%	56.6%	*	54.0%
Poverty status, % below poverty	47.3%	32.1%	***	42.0%
Education			***	
% < High school	32.6%	33.3%		32.9%
% High school diploma/GED	36.1%	28.5%		33.5%
% > High school	31.2%	38.2%		33.7%
Racial discrimination	1.77 (±1.92)	0.55 (±1.17)	***	1.34 (±1.79)
Sources of discrimination	18.43 (±5.79)	16.12 (±4.70)	***	17.63 (±5.55)
Lifetime discrimination burden	3.95 (±1.76)	3.15 (±1.65)	***	3.67 (±1.76)
Everyday discrimination	21.68 (±8.59)	21.03 (±7.99)	***	21.45 (±8.39)

*Note*. Racial group differences were examined with independent samples *t*-tests and chi-square tests of independence.

* *p* < .05

*** *p* < .001

We conducted race-stratified principal component analyses to assess the relationship among the four interpersonal discrimination indices used in this study (see S1 File). The results showed that a one-component (i.e., eigenvalue > 1) structure was the best fit for the data, explaining 60% and 57% of the shared variance for the four discrimination measures within African Americans and Whites, respectively. Further, Tucker’s coefficient of congruence revealed a high degree of congruence among the factor coefficients between African Americans and Whites (*r* = .89).

### Analyses with poverty status

**Fig 1:**
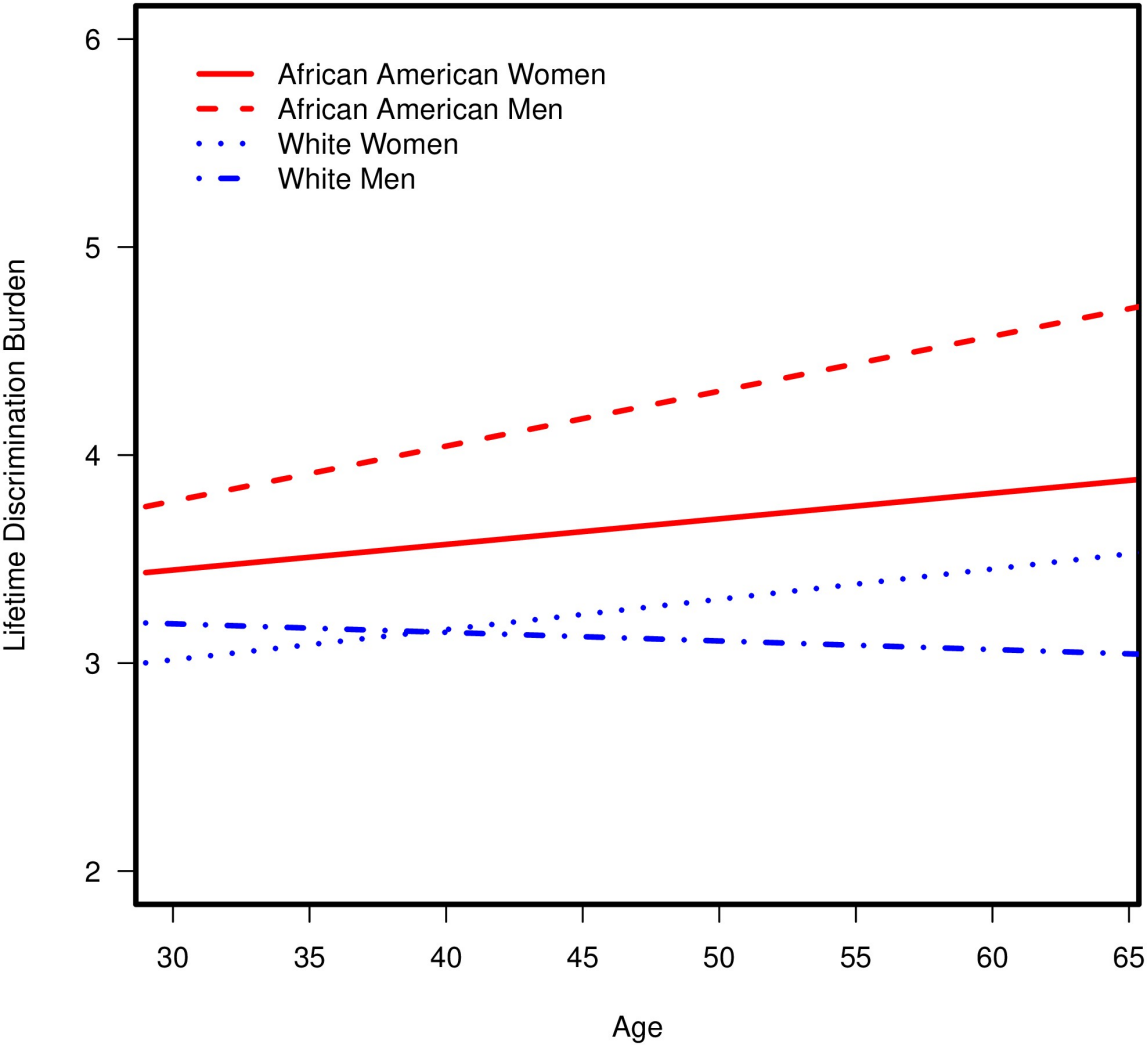
Significant three-way interaction of race × age × gender with lifetime discrimination burden.

Analyses with poverty status as the indicator of SES revealed no significant four-way interactions among race, age, gender, and poverty status. After backward elimination, models revealed two significant three-way interaction effects of (a) Race × Age × Gender with lifetime discrimination burden, *b* = 0.03, *p* = .019, and (b) Race × Gender × Poverty Status with lifetime discrimination burden, *b* = 0.57, *p* = 0.040 (Table 2). As shown in Fig 1, simple effects analyses revealed that older age was associated with significantly greater lifetime discrimination burden among African American men, *b* = 0.03, *p* < .001, and African American women, *b* = 0.02, *p* = .006, with a more pronounced effect in African American men than African American women.

**Table 2:** Inverse Gaussian regression model estimating three-way interaction effects among race and age, gender, or poverty status with lifetime discrimination burden.

Variable	*b*	*se*	*p*	95% CI	
				Lower	Upper
Race	0.12	0.54	.851	-0.95	1.15
Age	0.01	0.01	.215	-0.01	0.03
Gender	0.97	0.56	.087	-0.14	2.04
Poverty status	0.11	0.59	.867	-1.05	1.25
Race × Age	0.01	0.01	.430	-0.01	0.03
Race × Gender	-1.11	0.70	.112	-2.48	0.26
Race × Poverty Status	0.79	0.73	.270	-0.62	2.21
Age × Gender	-0.02	0.01	.100	-0.04	0.00
Age × Poverty Status	0.01	0.01	.490	-0.02	0.03
Gender × Poverty Status	-0.43	0.23	.058	-0.89	0.01
Race × Age × Gender	0.03	0.01	.019	0.01	0.06
Race × Age × Poverty Status	-0.02	0.02	.140	-0.05	0.01
Race × Gender × Poverty Status	0.57	0.28	.040	0.03	1.11

**Fig 2:**
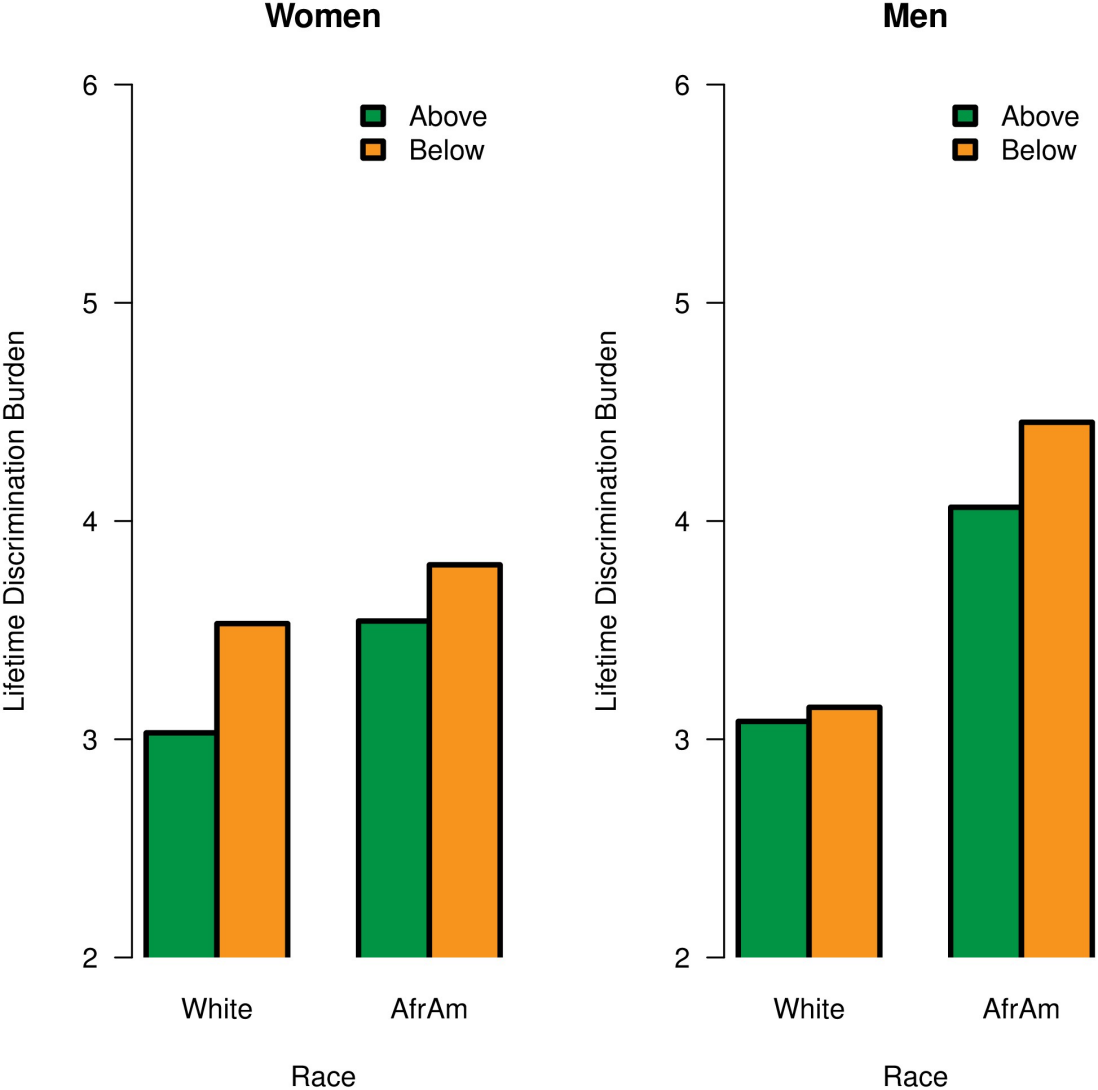
Significant three-way interaction of race × gender × poverty status with lifetime discrimination burden.

Next, as shown in Fig 2, simple effects analyses also revealed that living in poverty (versus living above poverty) was associated with significantly greater lifetime discrimination burden among African American men, *b* = 1.03, *p* = .014, and African American women, *b* = 0.90, *p* = .033.

Further, the association between living in poverty and greater lifetime discrimination burden was stronger among African American men than African American women. In contrast, associations between age and poverty status with lifetime discrimination burden were nonsignificant among White men and women (*p*’s >.05).

**Fig 3:**
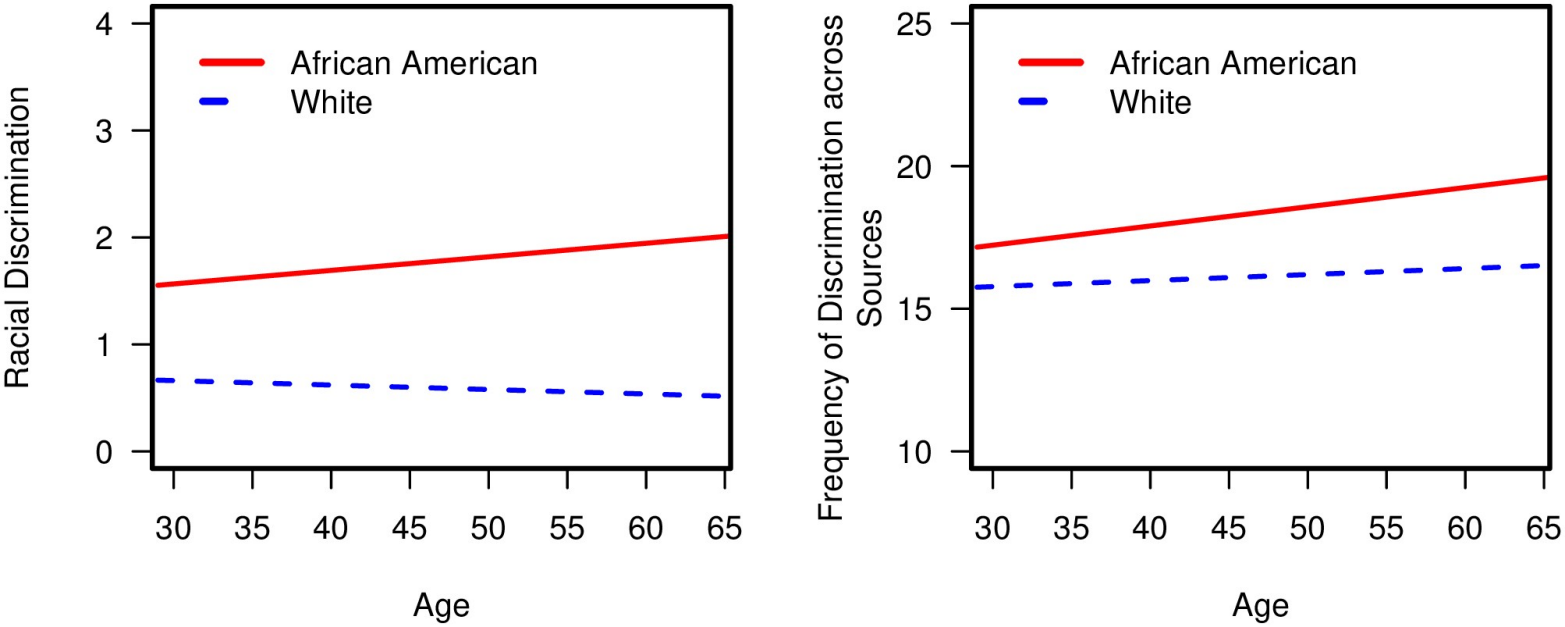
Significant two-way interactions of race × age with (a) racial discrimination and (b) frequency of discrimination across sources.

There were no further significant three-way interactions for the other discrimination indices, thus we backward eliminated. Subsequent analyses revealed four significant two-way interaction effects (Table 3). Specifically, there were two significant two-way interactions of Race × Age with (a) racial discrimination, *b* = 0.02, *p* = .014, and (b) frequency of discrimination across sources, *b* = 0.05, *p* = .036. As demonstrated in Fig 3, among African Americans, older age was associated with greater (a) racial discrimination, *b* = 0.01, *p* = .002, and (b) frequency of discrimination across sources, b = 0.07, p < .001.

**Table 3:** Inverse Gaussian regression model estimating two-way interaction effects among race and age, gender, or poverty status with multiple indices of discrimination.

**(a) Racial discrimination**					
Variable	*b*	*se*	*p*	95% CI	
				Lower	Upper
Race	0.16	0.35	.653	-0.52	0.84
Age	-0.00	0.01	.447	-0.02	0.01
Gender	0.03	0.11	.754	-0.17	0.24
Poverty status	0.23	0.11	.043	0.01	0.45
Race × Age	0.02	0.01	.014	0.003	0.03
Race × Gender	0.59	0.13	< .001	0.34	0.84
Race × Poverty Status	-0.10	0.14	.441	-0.37	0.16
**(b) Frequency of discrimination across sources**					
Variable	*b*	*se*	*p*	95% CI	
				Lower	Upper
Race	-0.79	0.12	.481	-2.98	1.40
Age	0.02	0.02	.233	-0.01	0.06
Gender	-1.06	0.34	.002	-1.73	-0.40
Poverty status	0.56	0.36	.123	-0.15	1.26
Race × Age	0.05	0.02	.036	0.003	0.09
Race × Gender	1.79	0.42	< .001	0.96	2.61
Race × Poverty Status	-0.09	0.44	.844	-0.94	0.77
**(c) Everyday discrimination**					
Variable	*b*	*se*	*p*	95% CI	
				Lower	Upper
Race	-1.84	1.71	.282	-5.19	1.51
Age	-0.17	0.03	< .001	-0.22	0.12
Gender	0.34	0.52	.511	-0.67	1.36
Poverty status	0.56	0.55	.312	-0.52	1.63
Race × Age	0.04	0.03	.038	-0.03	0.10
Race × Gender	1.33	0.64	.274	0.08	2.59
Race × Poverty Status	0.08	0.67	.905	-1.23	1.39

**Fig 4:**
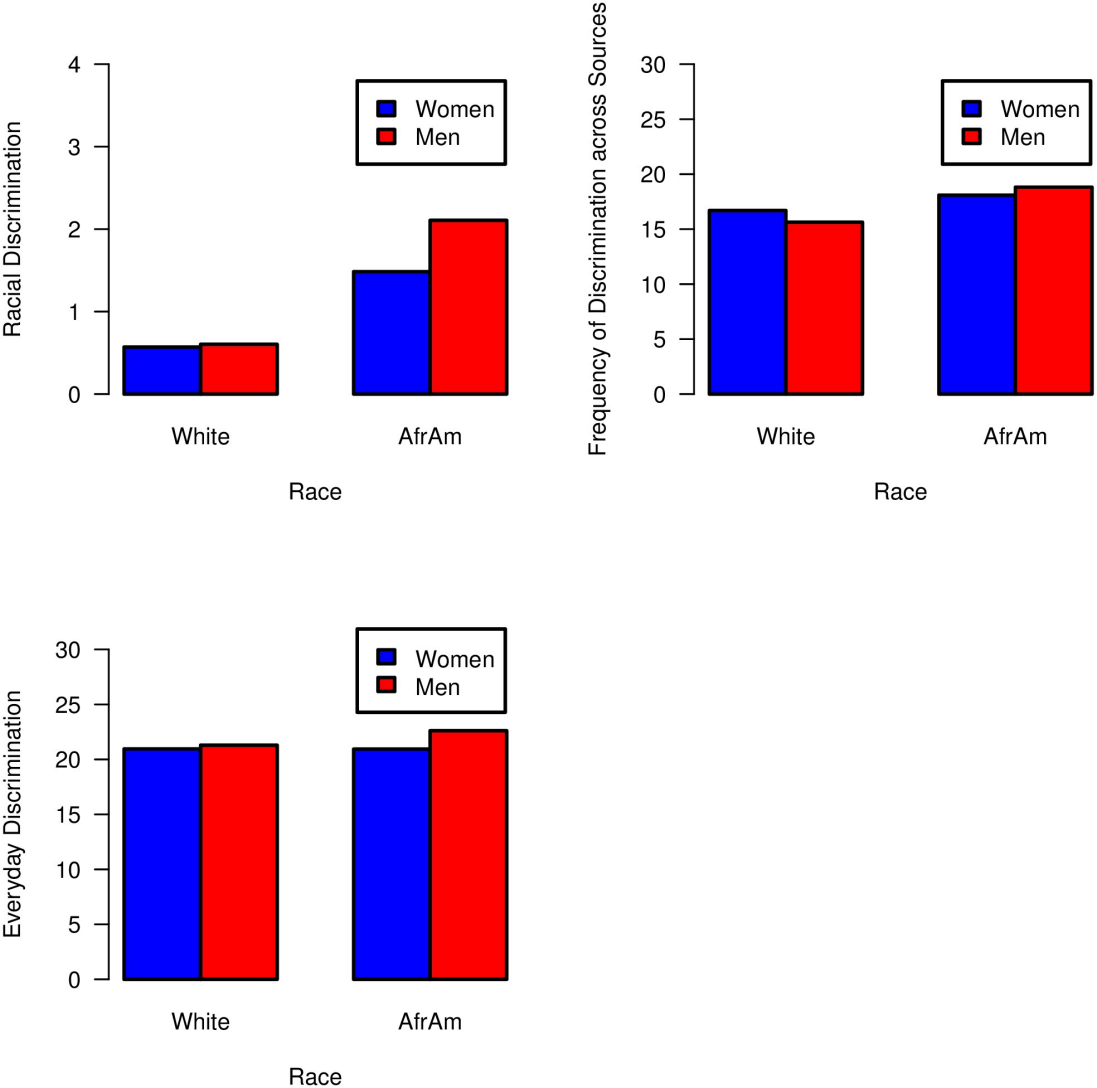
Significant two-way interactions of race × gender with (a) racial discrimination, (b) frequency of discrimination across sources, and (c) everyday discrimination.

In contrast, age was not associated with these discrimination indices among Whites (*p*’s >.05). Next, there were three significant two-way interactions of Race × Gender with (a) racial discrimination, *b* = 0.59, *p* < .001, (b) frequency of discrimination across sources, *b* = 1.79, *p* < .001, and (c) everyday discrimination, *b* = 1.33, *p* = .038. As demonstrated in Fig 4, African American men (versus African American women) reported significantly greater (a) racial discrimination, *b* = 0.62, *p* < .001, (b) frequency of discrimination across sources, *b* = 0.72, *p* = .003, and (c) everyday discrimination, *b* = 1.67, *p* < .001.

In contrast, White women reported significantly greater frequency of discrimination across sources than White men, *b* = -1.06, *p* = .002; no other significant differences were found between White men and women (*p*’s >.05).

### Analyses with education

Secondary analyses that included education as the indicator of SES (in lieu of poverty status) also revealed no significant four-way interaction effects among race, age, gender, and education with any of the discrimination measures. After backward elimination, analyses revealed one significant three-way interaction effect (S2 Table). Specifically, the previously observed three-way interaction of Race × Age × Gender with lifetime discrimination burden remained significant with education was substituted for poverty status in the model, *b* = 0.04, *p* = .012. As was observed previously, greater age was associated with greater lifetime discrimination burden among African American men, *b* = 0.03, *p* < .001 (S1 Fig).

However, the association between age and lifetime discrimination burden among African American women was nonsignificant when education was substituted for poverty status in the model, *b* = 0.01, *p* = .055. Associations between age and lifetime discrimination burden among White men and women remained nonsignificant when education was substituted for poverty status (*p*’s >.05).

There were no further significant three-way interactions for the other discrimination indices, therefore we backward eliminated. As in the models with poverty status, analyses adjusting for education revealed significant two-way interactions of Race × Age with racial discrimination and frequency of discrimination across sources (*p*’s < .05), such that older age was associated with greater discrimination on these measures among African Americans but not Whites (see S3 Table and S2 Fig). Further, as was observed in the primary models with poverty status, analyses adjusting for education revealed significant two-way interactions of Race × Gender with racial discrimination, frequency of discrimination across sources, and everyday discrimination (*p*’s < .05; see S3 Table and S2 Fig).

Additionally, these analyses revealed three new significant two-way interactions of Race × Education for (a) racial discrimination, *b* = 0.27, *p* = .001, (b) frequency of discrimination across sources, *b* = 0.64, *p* = .011, and (c) everyday discrimination, *b* = 1.16, *p* = .003 (see S3 Table). Among African Americans, greater educational attainment was associated with greater racial discrimination, *b* = 0.20, *p* < .001, and frequency of discrimination across sources, *b* = 0.41, *p* = .001 (S4 Fig); these effects were not observed among Whites (*p*’s >.05). Finally, among Whites, lower educational attainment was associated with greater everyday discrimination, *b* = -0.82, *p* = .007, whereas no association was found among African Americans, *b* = 0.34, *p* = .153 (see S4 Fig).

## Discussion

We examined patterns of multiple dimensions of interpersonal-level discrimination across key social statuses—race, age, gender, and SES—in an urban population of middle-aged to older African American and White adults. Our conceptual framework integrated elements from several relevant approaches including the racism-related stress framework [1], social stress theory [116, 117], and the intersectionality framework [93, 94]. Broadly, we observed that African Americans experienced all forms of discrimination more frequently than Whites, but this relationship was qualified by interactions of race with age, gender, and/or poverty status. In our study population, older African Americans, particularly older African American men, and African American men living in poverty, reported the greatest lifetime discrimination burden. Older African Americans also reported greater racial discrimination and greater frequency of social status-based discrimination than younger African Americans. African American men reported greater racial and everyday discrimination and a higher frequency of social status-based discrimination than African American women. We also observed that White women reported a greater frequency of discrimination than White men. Our findings show how race alongside age, gender, and SES shape experiences of interpersonal-level discrimination across two racial groups with divergent histories and contemporary lived experiences in the U.S.

### Patterning of discrimination

#### Race and age

A predominant theme in the current study demonstrated interactive patterning of race with age, such that older African Americans reported a greater frequency of discrimination across multiple social statuses, racial discrimination, and everyday discrimination. These findings are consistent with some recent studies (e.g., [36, 118]) that suggest age may partially reflect the cumulative burden of discrimination among African Americans and serve as a valuable proxy for exposure to social adversity across the lifespan [37]. While previous results from studies regarding age and interpersonal-level discrimination in African Americans are equivocal [5–8], these inconsistencies might result from methodological differences, such as not using the most relevant timeframe to measure exposure (e.g., past 12 months versus past 3 years) or using unidimensional measures that limit a fuller examination of the range of discriminatory experiences that African Americans face. Our findings support the life course perspective of discrimination, which suggests that while age and time function as biological processes, they can also function as markers of social significance [36]. African Americans coming of age closer in time to the Civil Rights Movement would have experienced a social and cultural milieu greatly influenced by race and discriminatory practices. Almost a third (30%) of the current participants lived through the Civil Rights Movement era. Our results support that older African Americans have faced more discrimination coinciding with historical periods of entrenched de jure and ensuing de facto structural racism through which resources, opportunities for advancement, and equity in protection are obtained. Our findings suggest that a finer grained delineation of interpersonal-level discrimination for African Americans may require gauging the contribution of how variations in sociohistorical and contemporary racism may contribute to their lived experiences.

#### Race, age, and gender

We also observed an interactive relationship between race, age, and gender—chiefly that older African American men reported the greatest lifetime burden of discrimination. This pattern is consistent with a preponderance of prior research suggesting that African American men report more self-reported discrimination (overall and race-based discrimination; although a few exceptions exist, see [5] for a review) than African American women. However, our results indicate that the overall burden of all discriminatory experiences, including variations in experiences by race and gender, may be predicated in part upon age [37]. The intersectional paradox may be appropriate for contextualizing this pattern. Specifically, in alignment with the hierarchy of sociodemographic categories in the U.S., African American men represent a social threat [64–66], because they occupy a minority sociodemographic (race), as well as a majority sociodemographic position of advantage (gender). As a result, their ideal superordinate category (gender) status may confer certain benefits, while their subgroup status as African Americans confers risks. Specifically, the primacy of their maleness in U.S. society, which *can* (or should) provide assurances of privilege and freedom in a patriarchal culture, is juxtaposed with their (highly stigmatized) race, which may invoke stereotypical depictions of them as violent, threatening, and listless [67, 119]. Indeed, African American men have been and continue to be key targets of race-based mistreatment, degradation, and violence in the U.S. (e.g., [58]). Further, older age may allow for greater cumulative exposure to discrimination as well as the effects of ageism per se [120].

Of course, it is difficult to tease apart the influence of age from other relevant factors, including historical period. Older African Americans in our sample would have come of age during the mid-1950s to 1970s when the Civil Rights Movement and de facto discrimination and racism would have been major aspects of their everyday lives. Our assessment of lifetime discrimination captured how much discrimination interfered with their ability to have a full and productive life, and how much harder it made their lives. It is plausible that the combination of the race-related transitions impacting all aspects of society and the related challenges they faced during this particular period, alongside their race and gender, left an indelible impression on their life course, shaping their opportunities and, consequently, their achievements, in ways that had cascading effects across their lives, creating a marked burden of discrimination. Indeed, our results are further supported by scores of narratives documenting the tumultuous and trying nature of this historical period for African American men (e.g., [121–123]). In sum, our findings may shed a critical light on the intersecting nature of race, age, and gender for older African American men in the context of historical discrimination. Future work exploring their life course narratives would help to further elucidate these linkages and their lived experiences of their sociodemographic memberships.

#### Race and gender

African American men reported greater frequency of discrimination across multiple social statuses, racial discrimination, and everyday discrimination than African American women. As discussed above, these results are largely consistent with the predominant finding across the literature that African American men report greater discrimination than African American women [5]. For instance, in an epidemiological study of 4,452 African Americans, men were more likely to report everyday and racial discrimination than women [124]. However, researchers have suggested that the greater discrimination burden reported by African American men is driven by a higher prevalence of racist experiences among men, a greater willingness of men to affirm these experiences, or from measurement challenges wherein discriminatory experiences gendered as female are not as adequately captured [55]. Indeed, for African Americans, the complex patterning of discrimination when race and gender are simultaneously considered raises questions about the nature of discriminatory experiences of African American women ([20, 21]). The current findings largely demonstrate that when the experience of discrimination is qualified by gender, African American men report more of these experiences. Qualitative evidence suggests that the lower prevalence of self-reported discrimination in African American women may not reflect an actual lower frequency of discriminatory experiences, but rather, differences in the types of discrimination they encounter across specific domains [i.e., 55; for further discussion see, 20, 21] and limited assessments of discrimination occurring at the intersection of their race and gender. For example, while African American men may be more likely to experience “criminal profiling” characterized by unwarranted police encounters based on unfounded judgments that they are physical threats, African American women may face “interpersonal incivilities” characterized by subtle expressions of disrespect, inappropriate breaches of social boundaries or attention, or being treated as invisible [55]. Thus, the discrimination burden of African American women may simply be comprised of *different* discriminatory experiences that may be as common or as great in magnitude as those experienced by African American men. In addition, African American women are doubly marginalized, as explained by the concept of intersectional invisibility [125]: African American women are completely marginalized in relation to the combination of their African American and gender [63, 125]. Therefore, African American women face both interracial and cross-racial sexism, as demonstrated in emerging empirical research (e.g., [20, 21]) and anecdotal and cultural accounts (e.g., [126]) that may not be well assessed in traditional racial or gender discrimination measures. Future research should examine gendered racism specific to the sociohistorical context that disparately shapes the experiences of African American men and women.

We found only one significant relationship for Whites in relation to discrimination—gender. White women reported a significantly greater frequency of discrimination across social statuses than White men; approximately 60% of White women identified their gender as a source of discrimination, followed by income and appearance. Thus, while White women still experience sexism, neither race, age, nor SES predicted their experiences with discrimination. These findings further bolster the intersectionality paradox, as some of White women’s statuses may serve as protective (e.g., race), while others may allow for discriminatory interactions (e.g., gender). Furthermore, these findings may partially reflect the longstanding, delicate connection between White women and African Americans in their struggles for equality. The Abolitionist Movement and the subsequent Women’s Suffrage Movement fought for equality with respective emphases on race and gender as single minority social statuses. A prevailing tension-invoking sentiment was that if African American men were to have freedom, surely White women should at the least possess liberties and civic participatory rights [absent consideration of African American women; 64, 127]. Consequently, White women’s experiences with gender discrimination, but not with racial discrimination, align with the sociohistorical lineage of these statuses in the U.S.

#### Race, gender, and SES

Our results illustrated how poverty and education are differentially associated with discrimination for African Americans as compared with Whites. When the role of poverty was considered, we observed that African Americans living in poverty (versus above poverty) reported greater lifetime discrimination burden, an effect that was more pronounced among African American men than African American women. The pronounced effect observed in African American men may reflect their distinct, intersecting statuses in American society. African American men continue to face a unique set of obstacles as related to SES and their lived experience of race-related adversity in the U.S. Compared with African American women and White men or women, African American men have much lower employment and high school completion rates and higher incarceration rates [128] and relatedly, have worse trajectories for income, wealth, and education—core conduits for disrupting poverty. These gender-by-race disparities may be underscored by perceptions of African American males as threatening, criminal, and violent. Indeed, these perceptions emerge in childhood, contributing to harsher and more punitive reactions from their teachers [129]. These perceptions also are linked to more adverse consequences during interactions with law enforcement and the criminal justice system—shown by their increased risk of being killed by law enforcement and their receiving unfair trials and harsher sentences [130, 131]. The collective impact of these types of race-related adversities on African American males may stymie their ability to rise from or avoid descending into poverty in adulthood and certainly impedes their ability to lead more fruitful lives overall. In sum, these factors may shed light on why the lifetime burden of discrimination is particularly meaningful for the lived experience of African American men living in poverty.

When we examined the role of education as the SES indicator, we observed some distinct patterns. For instance, greater educational attainment was related to greater discrimination exposure across social statuses for African Americans but not Whites, and lower educational attainment was related to greater everyday discrimination among Whites but not for African Americans. Our findings for African Americans are underscored by several theories including John Henryism [132], skin-deep resilience [133], and diminishing returns [134], which altogether assert that while African Americans may strive to attain higher levels of achievement, they may derive less benefit from attaining these outcomes. Higher educational attainment may not be commensurate with greater equity in interpersonal treatment for African Americans, but instead may exacerbate exposure to discrimination based on race and other social statuses they hold, which does not occur for Whites with similar achievements. African Americans may expect that they will have greater access to services and opportunities commensurate with their striving for upward mobility, in alignment with the American ideology that education is the “great equalizer” of differences and inequity [135]. Yet, our results echo those of other recent studies that suggest that achieving more appears to confer less benefit for African Americans and may be a source of risk: upwardly mobile African Americans have been found to experience more interpersonal discrimination than their SES-stable African American and White counterparts [136] or their African American peers with lower SES [17]. Our observation of greater discrimination across social status and race for African Americans suggests that there is something distinct about these social statuses as related to their educational attainment. For instance, it could suggest that African Americans with greater education are more likely to navigate predominately White settings or have interactions that yield more social status-based discrimination, especially racial discrimination [e.g., 137]. In this regard, our assessment of racial discrimination does capture these events across major life domains including, school, when seeking employment, housing, or medical care and at work, or in interactions with the police or legal system. Thusly, it is plausible that they are more likely to have access to more diverse settings as a result of their educational attainment, but yet face pushback based on their minority social memberships in those settings.

We observed that Whites with less education reported greater everyday discrimination than Whites with more education. Our findings suggest that attaining higher education serves a protective function for Whites, similar to Colen et al. [136] who found that as Whites realize greater upward SES mobility over time, they also experience less exposure to acute and chronic discrimination. In contrast to African Americans, Whites with more education in our study were more likely to affirm being treated with greater respect and courtesy than Whites with less education. This may demonstrate that experiences of feeling unfairly treated could—to some extent—hinge upon SES for both African Americans and Whites. These findings in Whites may suggest that class influences their perception of respect and fairness in interpersonal interactions, but do not suggest that their race bears upon these experiences.

In sum, these findings on the interactive role of race and SES, demonstrate at least three critical considerations. First, they provide strong support for the importance of investigating the intersectional linkages of race with other key sociodemographic factors, particularly SES, to further elucidate patterning of self-reported discrimination. Second, these findings demonstrate that experiences of and burdens associated with discrimination may be differentially linked to specific SES indicators and these linkages are not the same for Whites and African Americans. Different dimensions of SES may operate in unique ways [74] revealing variations in discriminatory experiences across key dimensions. Third, whereas Whites’ ascent up the SES ladder accrues better treatment, for African Americans lower and higher SES are both associated with greater discrimination risk [138]. This may suggest that there is never any relief for African Americans from discrimination whether or not they achieve greater SES.

### Further elucidation of multidimensional interpersonal discrimination

Although there is an emerging focus on discrimination as a multidimensional phenomenon [16–18], most prior research on interpersonal-level discrimination has either focused on a singular facet of discrimination or has only assessed discrimination among African Americans (e.g., Jackson Heart Study; [109]). However, when considered in aggregate, the above findings suggest that it may be important to study both race- and non-race-related experiences of discrimination in African Americans and Whites for several reasons.

One reason is that some discriminatory acts may not be explicitly race-related while other acts are. Individuals occupying multiple disadvantaged memberships may face discrimination associated not only with the respective minority memberships that they hold, but also across a combination of their disadvantaged and advantaged statuses [139]. In addition, simultaneously capturing multiple dimensions of discrimination may provide greater insight into the patterning of these experiences across various sociodemographic groupings, which may subsequently inform a host of other research areas [140].

Another factor to consider is that studies focusing on Whites’ self-reported experiences and their roles in conveying discrimination may be complementary to existing discrimination research. Specifically, examining the underlying conscious and unconscious psychological processes and the continuum on which discriminatory behavior is expressed (i.e., from subtle, non-race-related to blatantly race-related) may allow for a greater understanding of the rationale for White’s self-reports and their involvement in espousing discrimination. Also, in some discriminatory acts, the perpetrator or the target may be unaware that their experience is race-related, as factors such as personality, situational contexts, or unconscious Anti-Black bias can shape expressed behaviors [80, 141, 142].

Relatedly, it may be important to consider that differences across African Americans and Whites in their experiences of discrimination, particularly in the context of higher versus lower SES, may also bear upon cross-race empathy. That is, because Whites experience less discrimination and less burden overall, and particularly so as they gain higher education, they may not understand why discrimination (and perhaps other race-related adversity) are such a hindrance for African Americans, especially those who have also attained higher SES [143]. While Whites are also adversely affected by poverty, they are less so than African Americans. Research addressing these factors holds great promise for elucidating the cycle of discrimination that is commonplace in U.S. society.

### Strengths and limitations

A primary strength of this study is its inclusion of African Americans and Whites, as most studies of interpersonal-level discrimination have focused on racial or ethnic minorities, without surveying Whites on their perceptions of discrimination. We also examined whether multiple sociodemographic memberships might shape an individual’s experiences with discrimination, a little-explored avenue of research. In addition, we also included the interaction between race and SES in our analysis, which researchers have argued is valuable to examine, despite the inherent difficulty in disentangling the overlapping influences of each sociodemographic category [144]. Finally, building on a nascent area of discrimination research [17, 18], we considered multiple forms of interpersonal-level discrimination, while most past research has focused on a single form. Our approach allowed for a comprehensive investigation of the central question: how do experiences of interpersonal-level discrimination vary in accordance with intersections among race, age, gender, and SES?

As with all studies, our research had limitations. First, we did not evaluate SES indicators other than poverty and education; including other SES indicators (e.g., wealth and assets [47]) may further advance our understanding of the relationship of SES and interpersonal-level discrimination, especially in the context of race. Second, we surveyed only two racial groups; including other racial groups in this type of analysis would shed greater light on variations in the relationships between sociodemographic characteristics and interpersonal-level discrimination. In this regard, future studies should also explore the contribution of ethnicity within and between racial groups to shed further light on within racial group diversity and related differences in marginalization that may be specific to ethnicity, but not fully reflected by race. Additionally, given the continuous diversification of the American landscape, greater attention must be given biracial identities as well. Third, the present study examined self-identified gender (versus biological sex). The HANDLS study did not explicitly ask participants to self-identify their gender, thusly, except for three respondents who indicated to research staff that their self-identified gender differed from their biological sex, we were unable to determine whether all participants’ gender identities differed from their biological sex. Relatedly, we were also unable to examine non-binary gender identities or gender fluidity. A more inclusive characterization of gender in the HANDLS sample might have added other dimensions to our analysis. Fourth, although the present findings are not generalizable to the national population, independent demographic empirical analyses have demonstrated that this cohort is representative of populations from 14 U.S. cities with similar densities and racial distributions [145]. Fifth, the present analyses do not formally test for measurement invariance (e.g., through multigroup confirmatory factor analysis) to determine whether Whites and African Americans can be compared across dimensions of interpersonal discrimination. However, results from the race-stratified principal component analyses of the four interpersonal discrimination indices used in this study (see S1 File) and the calculation of Tucker’s coefficient of congruence determined that these indices are likely measuring similar underlying constructs in both racial groups. Finally, our results should be interpreted cautiously and viewed as preliminary given their overall exploratory nature and because we did not apply *p*-value adjustments for multiple comparisons.

### Future directions: Looking to health

Inherent in the statement by LeBron James was an awareness that discrimination is embedded *within* the structure of U.S. culture—a view widely held by theorists, historians, and researchers attentive to the implications of race as a social construct. To this end, the consequences of longstanding racial discrimination in the U.S. are apparent in well-documented racial inequities across core life domains, including those related to health, social relationships, education, civic and legal processes and institutions, and the economy [146]. In each of these domains, striking and protracted racial disparities in access, opportunities, resources, and welfare exist, in which African Americans fare more poorly than most other minority racial and ethnic groups, especially Whites. Understanding that these population-level, race-based patterns are *not* coincidental or the result of individual-level choices or behaviors [96] warrants that greater attention be given to the multilevel influences that support and reinforce individual-level discrimination processes.

Applying a socioecological perspective to elucidate the linkages among race, other sociodemographic categories, and the disproportionate burden of poor health on racial and ethnic minorities will help increase understanding of the full impact of discrimination on health. At least three considerations should be considered in future research. First, comprehensive investigation of the concurrent structural, cultural, individual, and intraindividual-level aspects of discrimination and racism is needed for a more inclusive picture of how these processes inform opportunities and well-being. Further, such work must engage the sociohistorical landscape underpinning these dynamics and examine the macro-level vestiges of de jure racism emerging as de facto racism [4]. For instance, recent studies drawing on interdisciplinary resources and methodologies have demonstrated that contemporary (1968–2014) heart disease mortality rates decline more slowly in U.S. counties with stronger legacies of African American slavery [147]; nationwide police killings of unarmed African Americans significantly impact mental health for African Americans but not Whites [148]; and neighborhood desirability ratings based on race and other minority sociodemographic categories from almost a century ago predict present day greater neighborhood violence risk in urban U.S. settings [149]. Altogether, these studies illustrate the relevance of historical and structural racism in shaping experiences at the interpersonal level [150] and highlight the need for more descriptive research to explicate linkages among intersecting minority sociodemographic categories and multilevel- and multidimensional-discrimination to better understand the intergenerational transmission of health inequity across a myriad of endpoints.

Second, emphasis on understanding more nuanced and ambiguous interpersonal-level discriminatory experiences, as well as vicarious, cultural-, and media-related experiences, should be considered as they may reflect the modern zeitgeist and current events [1] shaping day-to-day life. Indeed, the current findings provide strong support for such future work as we demonstrate that middle-aged to older African Americans—in a single cohort—are having diverse experiences with interpersonal-level discrimination across key forms—including racial and everyday discrimination, discrimination across multiple social statuses, and lifetime burden of discrimination. Also, the current report highlights the variability of these discriminatory events, showing their interactive linkages with other relevant sociodemographic categories, in addition to race. These findings help to draw needed attention to the complexity of the *lived* experience of discrimination. Therefore, this work points to the need for future empirical investigations which not only assess *better-established* forms of discrimination (e.g., racial and everyday), concurrently, but also more fully engages a multidimensional framework, wherein a broader array of discriminatory experiences is examined (e.g., vicarious race-related and vicarious-everyday). Altogether, this would add greatly to our understanding of cumulative exposure to discrimination as well.

Finally, other sociodemographic memberships are also shaped by the broader socioecological context and the related social hierarchy, and a more complete engagement of them (e.g., sexual- and gender-related minority statuses, ableness) and how they comprise an individual’s identity is necessary, if not essential, in health disparities research. Future research should give greater attention to nuanced intersectional paradoxes for particular groups (e.g., higher SES African Americans; [151]) and intersectional invisibilities for others (e.g., higher SES, minority race, and physical ableness), alongside more commonly assessed sociodemographic categories (e.g., gender). In sum, a comprehensive research approach which considers the sociohistorical lineage of sociodemographic categories, discrimination, and racism, alongside their present-day enactments at each level of society and their collective production of inequity across the key domains of American life, is needed for all, as LeBron James stated, to “feel equal in America” [12].

## Supporting information

S1 Fig Significant three-way interaction of race × age × gender with lifetime discrimination in analyses with education. (PDF)

S2 Fig Significant two-way interactions of race × age with (a) racial discrimination and (b) frequency of discrimination across sources in analyses with education. (PDF)

S3 Fig Significant two-way interactions of race × gender with (a) racial discrimination, (b) frequency of discrimination across sources, and (c) everyday discrimination in analyses with education. (PDF)

S4 Fig Significant two-way interactions of race × education with (a) racial discrimination, (b) frequency of discrimination across sources, and (c) everyday discrimination in analyses with education. (PDF)

S5 Fig Significant two-way interaction of gender × education with lifetime discrimination burden among non-Hispanic participants. (PDF)

S1 File Supplementary principal component analysis. (DOCX)

S2 File Supplementary methods and results. (DOCX)

S1 Table Bivariate correlations among discrimination measures. (DOCX)

S2 Table Inverse Gaussian regression model estimating three-way interactions among race and age, gender, or education with lifetime discrimination burden. (DOCX)

S3 Table Inverse Gaussian regression models estimating two-way interactions among race and age, gender, or education with racial discrimination, frequency of discrimination across sources, and everyday discrimination. (DOCX)

S4 Table Inverse Gaussian regression model estimating three-way interaction effects among race and age, gender, or poverty status with lifetime discrimination burden after excluding Hispanic Whites. (DOCX)

S5 Table Inverse Gaussian regression model estimating two-way interaction effects among race and age, gender, or poverty status with multiple indices of discrimination after excluding Hispanic Whites. (DOCX)

S6 Table Inverse Gaussian regression model estimating three-way interactions among race and age, gender, or education with lifetime discrimination burden after excluding Hispanic Whites. (DOCX)

S7 Table Inverse Gaussian regression models estimating two-way interactions among race and age, gender, or education with racial discrimination, frequency of discrimination across sources, and everyday discrimination after excluding Hispanic Whites. (DOCX)
