# Supplementary figures and images for: Race and other sociodemographic categories are differentially linked to multiple dimensions of interpersonal-level discrimination: Implications for intersectional, health research
Source: PLoS One. 2021 May 19;16(5):e0251174. doi: 10.1371/journal.pone.0251174 (PMC8133471; doi:10.1371/journal.pone.0251174)

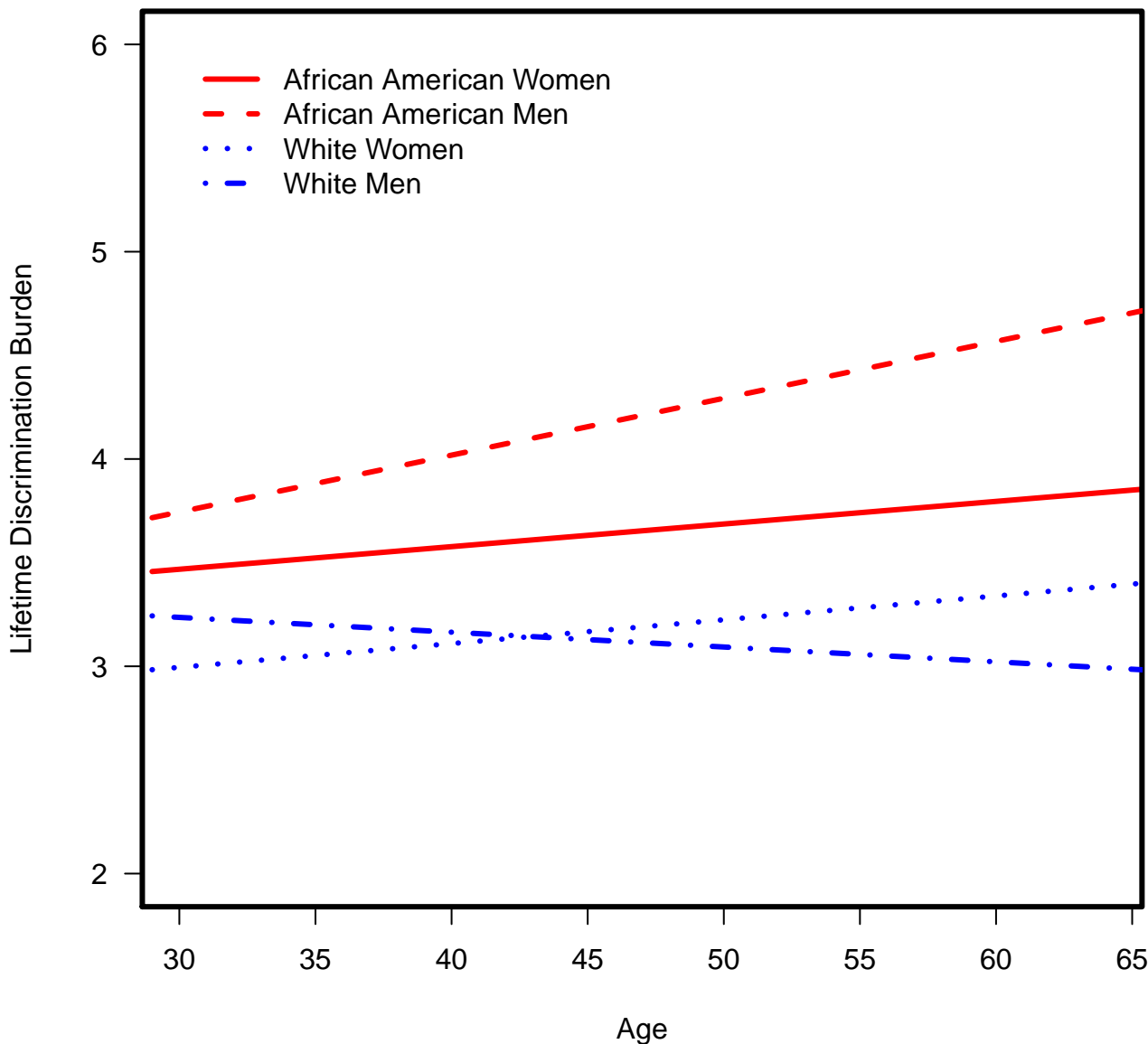

Supplement: S1 Fig — (PDF) [file pone.0251174.s001.pdf]

Racial Discrimination

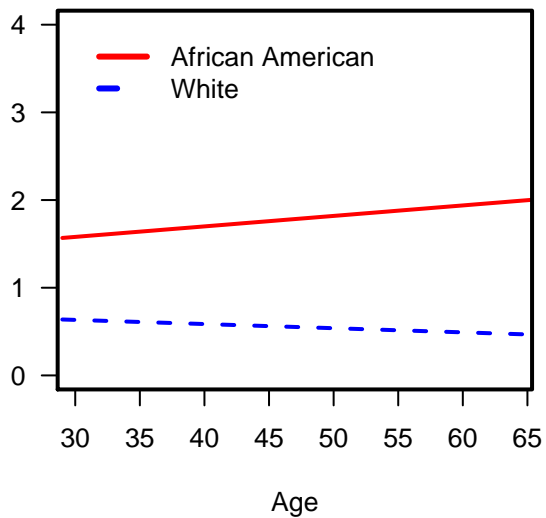

Frequency of Discrimination across Sources

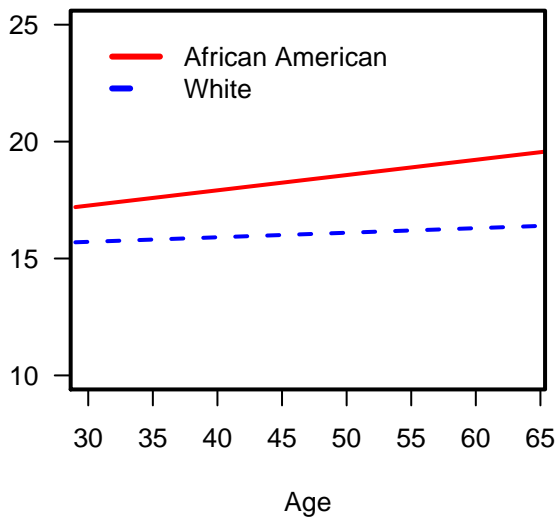

Supplement: S2 Fig — (PDF) [file pone.0251174.s002.pdf]

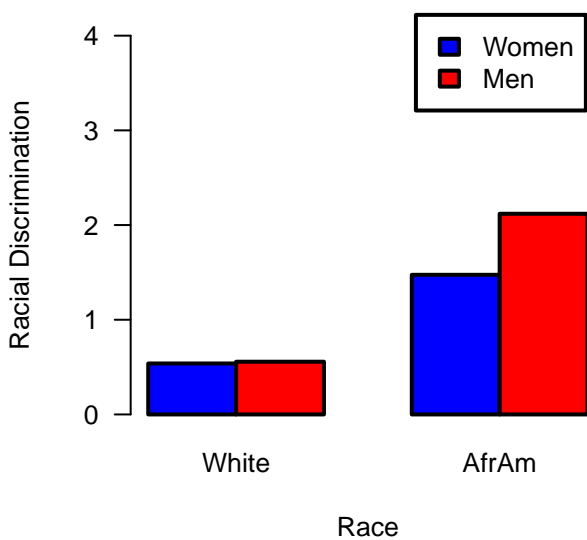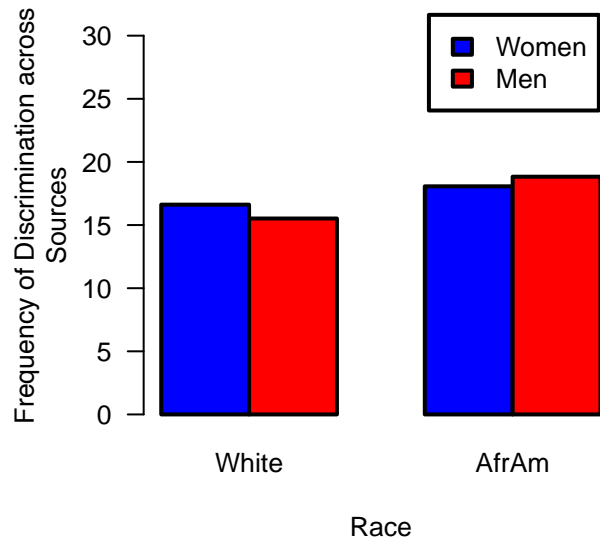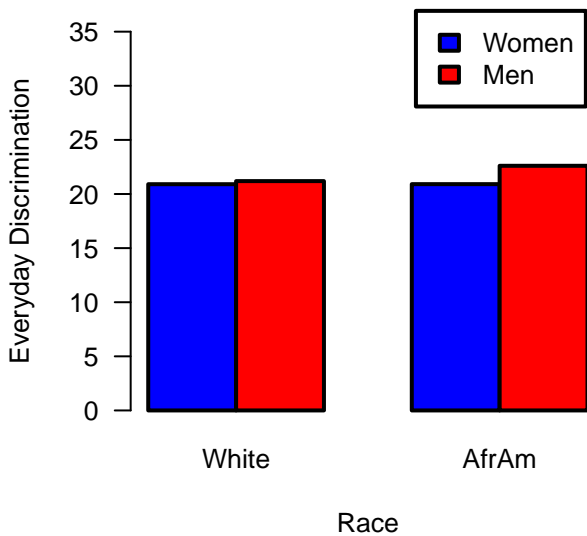

Supplement: S3 Fig — (PDF) [file pone.0251174.s003.pdf]

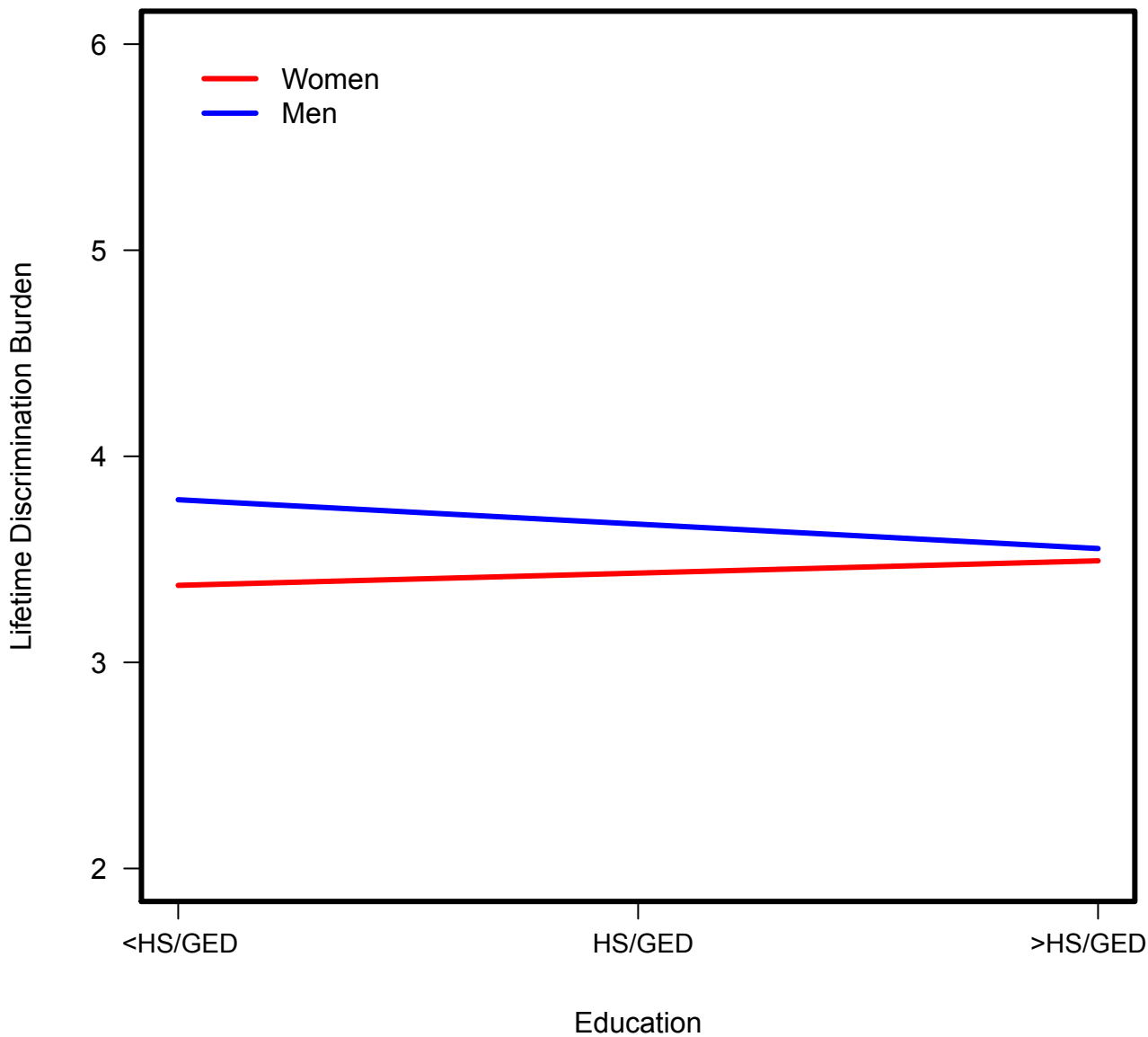

Supplement: S5 Fig — (PDF) [file pone.0251174.s005.pdf]
