# Supplementary material for: Race and other sociodemographic categories are differentially linked to multiple dimensions of interpersonal-level discrimination: Implications for intersectional, health research
Source: PLoS One. 2021 May 19;16(5):e0251174. doi: 10.1371/journal.pone.0251174 (PMC8133471; doi:10.1371/journal.pone.0251174)
Supplement: S4 Fig — (PDF) [file pone.0251174.s004.pdf]

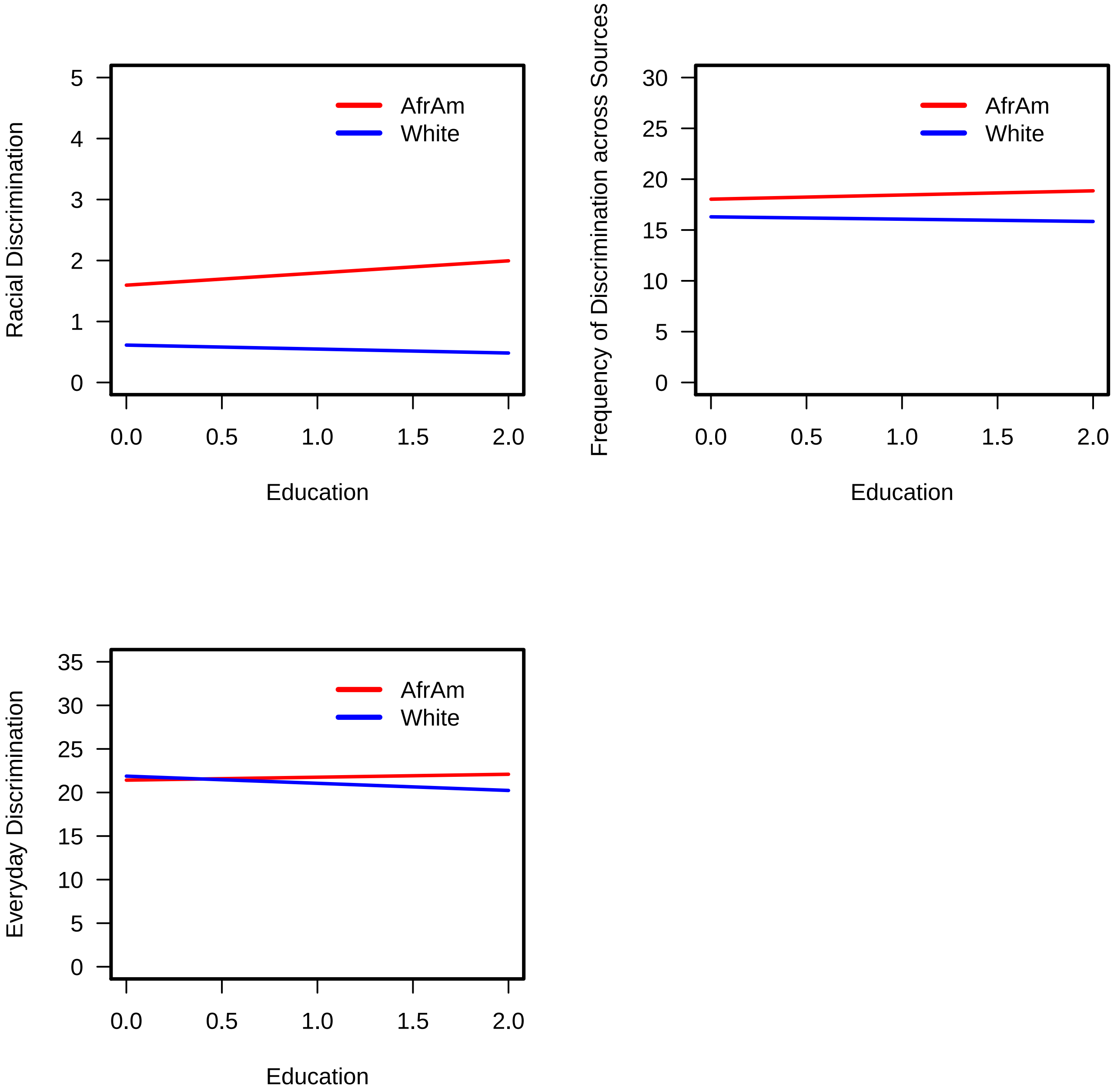

Racial Discrimination
Frequency of Discrimination across Sources
Everyday Discrimination
Education
AfrAm
White
0.0
0.5
1.0
1.5
2.0
0
1
2
3
4
5
10
15
20
25
30
35
