# Supplementary material for: Race and other sociodemographic categories are differentially linked to multiple dimensions of interpersonal-level discrimination: Implications for intersectional, health research
Source: PLoS One. 2021 May 19;16(5):e0251174. doi: 10.1371/journal.pone.0251174 (PMC8133471; doi:10.1371/journal.pone.0251174)
Supplement: S1 File — (DOCX) [file pone.0251174.s006.docx]

sort cases by race.

split file by race.

FACTOR

/VARIABLES disSources_nm disLifetime_nm disRacial_nm disEveryday_nm

/MISSING LISTWISE

/ANALYSIS disSources_nm disLifetime_nm disRacial_nm disEveryday_nm

/PRINT INITIAL EXTRACTION

/PLOT EIGEN

/CRITERIA MINEIGEN(1) ITERATE(25)

/EXTRACTION PC /ROTATION NOROTATE

/METHOD=CORRELATION.

# Factor Analysis

## Notes

| **Output Created** |  | **09-JUL-2020 09:44:24** |
| --- | --- | --- |
| **Comments** |  |  |
| **Input** | **Data** | **/Users/dleibel1/Box**  **Sync/Beatty**  **Lab/Patterns/Datasets/**  **R&R Datasets/Pat2.sav** |
|  | **Active Dataset** | **DataSet1** |
|  | **Filter** | **<none>** |
|  | **Weight** | **<none>** |
|  | **Split File** | **Race** |
|  | **N of Rows in Working Data File** | **2958** |
| **Missing Value Handling** | **Definition of Missing** | **MISSING=EXCLUDE: User-defined missing values are treated as missing.** |
|  | **Cases Used** | **LISTWISE: Statistics are based on cases with no missing values for any variable used.** |

## Notes

| **Syntax** |  | **FACTOR**  **/VARIABLES disSources_nm disLifetime_nm disRacial_nm disEveryday_nm /MISSING LISTWISE /ANALYSIS disSources_nm disLifetime_nm disRacial_nm disEveryday_nm**  **/PRINT INITIAL**  **EXTRACTION**  **/PLOT EIGEN**  **/CRITERIA MINEIGEN**  **(1) ITERATE(25)**  **/EXTRACTION PC**  **/ROTATION**  **NOROTATE**    **/METHOD=CORRELATIO**  **N.** |
| --- | --- | --- |
| **Resources** | **Processor Time** | **00:00:00.70** |
|  | **Elapsed Time** | **00:00:01.00** |
|  | **Maximum Memory Required** | **3008 (2.938K) bytes** |

# Race = White

**Communalities^a^**

|  | **Initial** | **Extraction** |
| --- | --- | --- |
| **disSources_nm** | **1.000** | **.653** |
| **disLifetime_nm** | **1.000** | **.641** |
| **disRacial_nm** | **1.000** | **.608** |
| **disEveryday_nm** | **1.000** | **.386** |

**Extraction Method: Principal Component Analysis.**

**a. Race = White**

## Total Variance Explained^a^

| **Component** | **Total** | **Initial Eigenvalues**  **% of Variance Cumulative %** | | **Extraction Sums of Squared Loadings**  **Total % of Variance Cumulative %** | | |
| --- | --- | --- | --- | --- | --- | --- |
| **1** | **2.289** | **57.220** | **57.220** | **2.289** | **57.220** | **57.220** |
| **2** | **.754** | **18.851** | **76.071** |  |  |  |
| **3** | **.518** | **12.949** | **89.020** |  |  |  |
| **4** | **.439** | **10.980** | **100.000** |  |  |  |

**Extraction Method: Principal Component Analysis.**

**a. Race = White**

**4**

**3**

**2**

**1**

**Eigenvalue**

**2.5**

**2.0**

**1.5**

**1.0**

**0.5**

**0.0**

**Race: White**

**Scree Plot**

**Component Number**

## Component Matrix ^a,b^

**Component 1**

| **disSources_nm** | **.808** |
| --- | --- |
| **disLifetime_nm** | **.801** |
| **disRacial_nm** | **.780** |
| **disEveryday_nm** | **.621** |

**Extraction Method: Principal Component Analysis.**

1. **Race = White**
2. **1 components extracted.**

# Race = AfrAm

## Communalities^a^

|  | **Initial** | **Extraction** |
| --- | --- | --- |
| **disSources_nm** | **1.000** | **.596** |
| **disLifetime_nm** | **1.000** | **.670** |
| **disRacial_nm** | **1.000** | **.661** |
| **disEveryday_nm** | **1.000** | **.484** |

**Extraction Method: Principal Component Analysis.**

**a. Race = AfrAm**

## Total Variance Explained^a^

| **Component** | **Total** | **Initial Eigenvalues**  **% of Variance Cumulative %** | | **Extraction Sums of Squared Loadings**  **Total % of Variance Cumulative %** | | |
| --- | --- | --- | --- | --- | --- | --- |
| **1** | **2.411** | **60.272** | **60.272** | **2.411** | **60.272** | **60.272** |
| **2** | **.707** | **17.679** | **77.951** |  |  |  |
| **3** | **.528** | **13.193** | **91.145** |  |  |  |
| **4** | **.354** | **8.855** | **100.000** |  |  |  |

**Extraction Method: Principal Component Analysis.**

**a. Race = AfrAm**

**4**

**3**

**2**

**1**

**Eigenvalue**

**2.5**

**2.0**

**1.5**

**1.0**

**0.5**

**0.0**

**Race: AfrAm**

**Scree Plot**

**Component Number**

## Component Matrix ^a,b^

**Component 1**

| **disSources_nm** | **.772** |
| --- | --- |
| **disLifetime_nm** | **.818** |
| **disRacial_nm** | **.813** |
| **disEveryday_nm** | **.696** |

**Extraction Method: Principal Component Analysis.**

1. **Race = AfrAm**
2. **1 components extracted.**
