# Supplementary material for: Race and other sociodemographic categories are differentially linked to multiple dimensions of interpersonal-level discrimination: Implications for intersectional, health research
Source: PLoS One. 2021 May 19;16(5):e0251174. doi: 10.1371/journal.pone.0251174 (PMC8133471; doi:10.1371/journal.pone.0251174)
Supplement: S2 File — (DOCX) [file pone.0251174.s007.docx]

**Supplementary Methods**

There were nine participants in the present study’s analysis sample who self-identified their ethnicity as Hispanic, all of whom self-identified their race as White. To determine whether Hispanic ethnicity influenced the results, sensitivity analyses were conducted by excluding these Hispanic White participants and rerunning all models. These sensitivity analyses were parallel to those conducted in the overall sample (see Statistical Plan subsection of the Methods in the main manuscript).

**Supplementary Results**

**Sensitivity Analyses**

All significant interactions and main effects previously found in the overall sample remained significant among Non-Hispanic participants (*p*’s < .05; see Supplementary Tables 4-7 for complete regression model results). In addition, there was a newly significant interaction of Gender × Education with lifetime discrimination burden, *b* = -0.26, *p* = .042 (see Supplementary Table 6). As shown in Supplemental Figure 5, among Non-Hispanic men, lesser educational attainment was associated with greater lifetime discrimination burden, *b* = -0.79, *p* = .019. In contrast, educational attainment was not associated with lifetime discrimination burden among Non-Hispanic women, *b* = -0.53, *p* = .104.

**Supplementary Figure 5.** Significant two-way interaction of Gender × Education with lifetime discrimination burden among Non-Hispanic participants.
