# Supplementary material for: Race and other sociodemographic categories are differentially linked to multiple dimensions of interpersonal-level discrimination: Implications for intersectional, health research
Source: PLoS One. 2021 May 19;16(5):e0251174. doi: 10.1371/journal.pone.0251174 (PMC8133471; doi:10.1371/journal.pone.0251174)
Supplement: S1 Table — (DOCX) [file pone.0251174.s008.docx]

| S1 Table. *Bivariate Correlations among Discrimination Measures* | | | | |
| --- | --- | --- | --- | --- |
|  | 1. | 2. | 3. | 4. |
| 1. Racial Discrimination | 1 | .48** | .62** | .37** |
| 1. Frequency of discrimination across sources |  | 1 | .53** | .44** |
| 1. Lifetime Discrimination Burden |  |  | 1 | .35** |
| 1. Everyday Discrimination |  |  |  | 1 |
| *Note*. ** *p* < .01 | | | | |
