# Supplementary material for: Race and other sociodemographic categories are differentially linked to multiple dimensions of interpersonal-level discrimination: Implications for intersectional, health research
Source: PLoS One. 2021 May 19;16(5):e0251174. doi: 10.1371/journal.pone.0251174 (PMC8133471; doi:10.1371/journal.pone.0251174)
Supplement: S2 Table — (DOCX) [file pone.0251174.s009.docx]

| S2 Table. *Inverse Gaussian Regression Model Estimating Three-way Interactions among Race and Age, Gender, or Education with Lifetime Discrimination Burden* | | | | | |
| --- | --- | --- | --- | --- | --- |
| Variable | *b* | *se* | *p* | 95% CI | |
|  |  |  |  | Lower | Upper |
| Race | 0.49 | 0.46 | .284 | -0.41 | 1.39 |
| Age | 0.01 | 0.01 | .122 | -0.00 | 0.03 |
| Gender | 0.80 | 0.55 | .146 | -0.28 | 1.88 |
| Education | -0.47 | 0.32 | .146 | -1.10 | 0.16 |
| Race × Age | -0.00 | 0.01 | .952 | -0.02 | 0.02 |
| Race × Gender | -1.02 | 0.69 | .138 | -2.37 | 0.33 |
| Race × Education | -0.10 | 0.42 | .817 | -0.91 | 0.72 |
| Age × Gender | -0.02 | 0.01 | .097 | -0.04 | 0.00 |
| Age × Education | 0.01 | 0.01 | .133 | -0.00 | 0.02 |
| Gender × Education | -0.24 | 0.13 | .061 | -0.49 | 0.01 |
| Race × Age × Gender | 0.04 | 0.01 | .012 | 0.01 | 0.06 |
| Race × Age × Education | 0.00 | 0.01 | .629 | -0.01 | 0.02 |
| Race × Gender × Education | 0.14 | 0.16 | .384 | -0.17 | 0.45 |
