# Supplementary material for: Race and other sociodemographic categories are differentially linked to multiple dimensions of interpersonal-level discrimination: Implications for intersectional, health research
Source: PLoS One. 2021 May 19;16(5):e0251174. doi: 10.1371/journal.pone.0251174 (PMC8133471; doi:10.1371/journal.pone.0251174)
Supplement: S3 Table — (DOCX) [file pone.0251174.s010.docx]

| S3 Table. *Inverse Gaussian Regression Models Estimating Two-way Interactions among Race and Age, Gender, or Education with Racial Discrimination, Frequency of Discrimination across Sources, and Everyday Discrimination* | | | | | |
| --- | --- | --- | --- | --- | --- |
| (a) Racial discrimination | | | | | |
| Variable | *b* | *se* | *p* | 95% CI | |
|  |  |  |  | Lower | Upper |
| Race | 0.13 | 0.34 | .696 | -0.53 | 0.80 |
| Age | -0.01 | 0.01 | .387 | -0.02 | 0.01 |
| Gender | 0.02 | 0.11 | .859 | -0.19 | 0.23 |
| Education | -0.07 | 0.06 | .291 | -0.19 | 0.06 |
| Race × Age | 0.02 | 0.01 | .015 | 0.003 | 0.03 |
| Race × Gender | 0.63 | 0.13 | <.001 | 0.37 | 0.88 |
| Race × Education | 0.27 | 0.08 | .001 | 0.11 | 0.42 |
| (b) Frequency of discrimination across sources | | | | | |
| Variable | *b* | *se* | *p* | 95% CI | |
|  |  |  |  | Lower | Upper |
| Race | -0.75 | 1.10 | .493 | -2.90 | 1.40 |
| Age | 0.02 | 0.02 | .270 | -0.02 | 0.05 |
| Gender | -1.10 | 0.34 | .001 | -1.76 | -0.44 |
| Education | -0.23 | 0.20 | .250 | -0.62 | 0.16 |
| Race × Age | 0.05 | 0.03 | .038 | 0.003 | 0.09 |
| Race × Gender | 1.86 | 0.42 | <.001 | 1.04 | 2.68 |
| Race × Education | 0.64 | 0.25 | .011 | 0.15 | 1.12 |
| (c) Everyday discrimination | | | | | |
| Variable | *b* | *se* | *p* | 95% CI | |
|  |  |  |  | Lower | Upper |
| Race | -1.81 | 1.68 | .280 | -5.09 | 1.47 |
| Age | -0.18 | 0.03 | <.001 | -0.23 | -0.12 |
| Gender | 0.29 | 0.52 | .581 | -0.73 | 1.30 |
| Education | -0.82 | 0.30 | .007 | -1.42 | -0.23 |
| Race × Age | 0.04 | 0.03 | .264 | -0.03 | 0.10 |
| Race × Gender | 1.41 | 0.64 | .028 | 0.16 | 2.66 |
| Race × Education | 1.16 | 0.38 | .003 | 0.40 | 1.91 |
