# Supplementary material for: Race and other sociodemographic categories are differentially linked to multiple dimensions of interpersonal-level discrimination: Implications for intersectional, health research
Source: PLoS One. 2021 May 19;16(5):e0251174. doi: 10.1371/journal.pone.0251174 (PMC8133471; doi:10.1371/journal.pone.0251174)
Supplement: S4 Table — (DOCX) [file pone.0251174.s011.docx]

| S4 Table. *Inverse Gaussian Regression Model Estimating Three-way Interaction Effects among Race and Age, Gender, or Poverty Status with Lifetime Discrimination Burden after Excluding Hispanic Whites* | | | | | |
| --- | --- | --- | --- | --- | --- |
| Variable | *b* | *se* | *p* | 95% CI | |
|  |  |  |  | Lower | Upper |
| Race | 0.06 | 0.54 | .907 | -0.99 | 1.12 |
| Age | 0.01 | 0.01 | .251 | -0.01 | 0.03 |
| Gender | 0.92 | 0.56 | .102 | -0.18 | 2.01 |
| Poverty status | 0.10 | 0.59 | .865 | -1.06 | 1.26 |
| Race × Age | 0.01 | 0.01 | .392 | -0.01 | 0.03 |
| Race × Gender | -1.07 | 0.70 | .126 | -2.45 | 0.30 |
| Race × Poverty Status | 0.80 | 0.73 | .273 | -0.63 | 2.22 |
| Age × Gender | -0.02 | 0.01 | .108 | -0.04 | 0.00 |
| Age × Poverty Status | 0.01 | 0.01 | .497 | -0.02 | 0.03 |
| Gender × Poverty Status | -0.43 | 0.23 | .063 | -0.88 | 0.02 |
| Race × Age × Gender | 0.03 | 0.01 | .022 | 0.01 | 0.06 |
| Race × Age × Poverty Status | -0.02 | 0.02 | .144 | -0.05 | 0.01 |
| Race × Gender × Poverty Status | 0.56 | 0.28 | .044 | 0.02 | 1.11 |
