# Supplementary material for: Race and other sociodemographic categories are differentially linked to multiple dimensions of interpersonal-level discrimination: Implications for intersectional, health research
Source: PLoS One. 2021 May 19;16(5):e0251174. doi: 10.1371/journal.pone.0251174 (PMC8133471; doi:10.1371/journal.pone.0251174)
Supplement: S5 Table — (DOCX) [file pone.0251174.s012.docx]

| S5 Table. *Inverse Gaussian Regression Model Estimating Two-way Interaction Effects among Race and Age, Gender, or Poverty Status with Multiple Indices of Discrimination after Excluding Hispanic Whites* | | | | | | |
| --- | --- | --- | --- | --- | --- | --- |
| (a) Racial discrimination | | | | | | |
| Variable | *b* | *se* | *p* | 95% CI | | |
|  |  |  |  | Lower | Upper | |
| Race | 0.19 | 0.35 | .588 | -0.49 | | 0.87 |
| Age | -0.00 | 0.01 | .539 | -0.01 | | 0.01 |
| Gender | 0.01 | 0.11 | .964 | -0.20 | | 0.21 |
| Poverty status | 0.22 | 0.11 | .049 | 0.001 | | 0.44 |
| Race × Age | 0.02 | 0.13 | .020 | 0.003 | | 0.03 |
| Race × Gender | 0.62 | 0.14 | <.001 | 0.36 | | 0.87 |
| Race × Poverty Status | -0.10 | 0.14 | .467 | -0.36 | | 0.17 |
| (b) Frequency of discrimination across sources | | | | | | |
| Variable | *b* | *se* | *p* | 95% CI | | |
|  |  |  |  | Lower | Upper | |
| Race | -0.84 | 1.12 | .453 | -3.03 | | 1.35 |
| Age | 0.02 | 0.02 | .264 | -0.02 | | 0.06 |
| Gender | -1.07 | 0.34 | .002 | -1.74 | | -0.41 |
| Poverty status | 0.51 | 0.36 | .160 | -0.20 | | 1.21 |
| Race × Age | 0.05 | 0.02 | .032 | 0.004 | | 0.09 |
| Race × Gender | 1.80 | 0.42 | <.001 | 0.98 | | 2.62 |
| Race × Poverty Status | -0.04 | 0.44 | .931 | -0.89 | | 0.82 |
| (c) Everyday discrimination | | | | | | |
| Variable | *b* | *se* | *p* | 95% CI | | |
|  |  |  |  | Lower | Upper | |
| Race | -1.92 | 1.71 | .263 | -5.28 | | 1.44 |
| Age | -0.17 | 0.03 | <.001 | -0.23 | | 0.12 |
| Gender | 0.30 | 0.52 | .569 | -0.72 | | 1.32 |
| Poverty status | 0.49 | 0.55 | .372 | -0.59 | | 1.58 |
| Race × Age | 0.04 | 0.03 | .264 | -0.03 | | 0.11 |
| Race × Gender | 1.38 | 0.64 | .032 | 0.12 | | 2.63 |
| Race × Poverty Status | 0.14 | 0.67 | .832 | -1.17 | | 1.45 |
