# Supplementary material for: Race and other sociodemographic categories are differentially linked to multiple dimensions of interpersonal-level discrimination: Implications for intersectional, health research
Source: PLoS One. 2021 May 19;16(5):e0251174. doi: 10.1371/journal.pone.0251174 (PMC8133471; doi:10.1371/journal.pone.0251174)
Supplement: S6 Table — (DOCX) [file pone.0251174.s013.docx]

| S6 Table. *Inverse Gaussian Regression Model Estimating Three-way Interactions among Race and Age, Gender, or Education with Lifetime Discrimination Burden after Excluding Hispanic Whites* | | | | | |
| --- | --- | --- | --- | --- | --- |
| Variable | *b* | *se* | *p* | 95% CI | |
|  |  |  |  | Lower | Upper |
| Race | 0.44 | 0.46 | .335 | -0.46 | 1.34 |
| Age | 0.01 | 0.01 | .161 | -0.00 | 0.03 |
| Gender | 0.75 | 0.55 | .174 | -0.33 | 1.84 |
| Education | -0.53 | 0.32 | .104 | -1.16 | 0.11 |
| Race × Age | 0.00 | 0.01 | .959 | -0.02 | 0.02 |
| Race × Gender | -0.97 | 0.69 | .159 | -2.32 | 0.38 |
| Race × Education | -0.04 | 0.42 | .927 | -0.86 | 0.78 |
| Age × Gender | -0.02 | 0.01 | .115 | -0.04 | 0.00 |
| Age × Education | 0.01 | 0.01 | .089 | -0.00 | 0.02 |
| Gender × Education | -0.26 | 0.13 | .042 | -0.51 | -0.01 |
| Race × Age × Gender | 0.03 | 0.01 | .015 | 0.01 | 0.06 |
| Race × Age × Education | 0.00 | 0.01 | .748 | -0.01 | 0.02 |
| Race × Gender × Education | 0.16 | 0.16 | .319 | -0.15 | 0.47 |
