# Supplementary material for: Race and other sociodemographic categories are differentially linked to multiple dimensions of interpersonal-level discrimination: Implications for intersectional, health research
Source: PLoS One. 2021 May 19;16(5):e0251174. doi: 10.1371/journal.pone.0251174 (PMC8133471; doi:10.1371/journal.pone.0251174)
Supplement: S7 Table — (DOCX) [file pone.0251174.s014.docx]

| S7 Table. *Inverse Gaussian Regression Models Estimating Two-way Interactions among Race and Age, Gender, or Education with Racial Discrimination, Frequency of Discrimination across Sources, and Everyday Discrimination after Excluding Hispanic Whites* | | | | | |
| --- | --- | --- | --- | --- | --- |
| (a) Racial discrimination | | | | | |
| Variable | *b* | *se* | *p* | 95% CI | |
|  |  |  |  | Lower | Upper |
| Race | 0.17 | 0.34 | .627 | -0.50 | 0.83 |
| Age | -0.00 | 0.01 | .469 | -0.02 | 0.01 |
| Gender | 0.01 | 0.11 | .928 | -0.22 | 0.20 |
| Education | -0.08 | 0.06 | .204 | -0.20 | 0.04 |
| Race × Age | 0.02 | 0.01 | .020 | 0.003 | 0.03 |
| Race × Gender | 0.65 | 0.13 | <.001 | 0.40 | 0.91 |
| Race × Education | 0.28 | 0.08 | <.001 | 0.13 | 0.43 |
| (b) Frequency of discrimination across sources | | | | | |
| Variable | *b* | *se* | *p* | 95% CI | |
|  |  |  |  | Lower | Upper |
| Race | -0.78 | 1.10 | .477 | -2.93 | 1.37 |
| Age | 0.02 | 0.02 | .301 | -0.02 | 0.05 |
| Gender | -1.11 | 0.34 | .001 | -1.44 | -0.44 |
| Education | -0.23 | 0.20 | .255 | -0.62 | 0.16 |
| Race × Age | 0.05 | 0.02 | .034 | 0.004 | 0.09 |
| Race × Gender | 1.87 | 0.42 | <.001 | 1.05 | 2.69 |
| Race × Education | 0.64 | 0.25 | .011 | 0.15 | 1.13 |
| (c) Everyday discrimination | | | | | |
| Variable | *b* | *se* | *p* | 95% CI | |
|  |  |  |  | Lower | Upper |
| Race | -1.86 | 1.68 | .268 | -5.15 | 1.43 |
| Age | -0.18 | 0.03 | <.001 | -0.23 | -0.12 |
| Gender | 0.24 | 0.52 | .646 | -0.78 | 1.26 |
| Education | -0.85 | 0.31 | .005 | -1.45 | -0.26 |
| Race × Age | 0.04 | 0.03 | .258 | -0.28 | 0.11 |
| Race × Gender | 1.46 | 0.64 | .023 | 0.20 | 2.72 |
| Race × Education | 1.18 | 0.39 | .002 | 0.43 | 1.94 |
